# Vascular smooth muscle cell–derived KIF13B inhibits proinflammatory responses to protect against atherosclerosis

**DOI:** 10.1172/JCI194175

**Published:** 2026-01-29

**Authors:** Guolin Miao, Yufei Han, Jingxuan Chen, Yiran Liu, Ge Zhang, Shaotong Pei, Yinqi Zhao, Yitong Xu, Liwen Zheng, Zhaoling Li, Xiangru Liu, Sijing Shi, Xuya Kang, Yahan Liu, Ling Zhang, Wei Huang, Yuhui Wang, Junnan Tang, Erdan Dong, Xunde Xian

**Affiliations:** 1 Department of Cardiology and Institute of Vascular Medicine, Peking University Third Hospital, Beijing, China.; 2 Institute of Cardiovascular Sciences, State Key Laboratory of Vascular Homeostasis and Remodeling, School of Basic Medical Sciences, and; 3 Department of Biomedical Informatics, School of Basic Medical Sciences, Peking University, Beijing, China.; 4 Department of Cardiology, The First Affiliated Hospital of Zhengzhou University, Zhengzhou, China.; 5 Research Center for Cardiopulmonary Rehabilitation, University of Health and Rehabilitation Sciences Qingdao Hospital (Qingdao Municipal Hospital), School of Health and Life Sciences, University of Health and Rehabilitation Sciences, Qingdao, China.; 6 Research Unit of Medical Science Research Management/Basic and Clinical Research of Metabolic Cardiovascular Diseases, Chinese Academy of Medical Sciences, Beijing, China.; 7 Department of Human Anatomy and Histology and Embryology, School of Basic Medical Sciences, Peking University, Beijing, China.; 8 Beijing Key Laboratory of Cardiovascular Receptors Research, Peking University Third Hospital, Beijing, China.

**Keywords:** Cardiology, Vascular biology, Atherosclerosis

## Abstract

Atherosclerotic cardiovascular disease (ASCVD) remains a leading cause of death worldwide, with plaque instability being a major culprit. Phenotypic switching of vascular smooth muscle cells (VSMCs) is a central event in atherosclerosis, driving both plaque progression and stability, yet the underlying mechanisms are incompletely understood, limiting drug development targeting this process. Kinesin family member 13B (KIF13B) has been implicated in vascular biology, but its function in VSMCs is unknown. Here, we demonstrate that VSMC-specific deletion of *Kif13b* in mice overexpressing proprotein convertase subtilisin/kexin type 9 (PCSK9) exacerbates lesion development and impairs plaque stability, characterized by thinner fibrous caps and increased inflammation. Mechanistically, we determined that KIF13B facilitated the ubiquitination and proteasomal degradation of Krüppel-like factor 4 (KLF4) through the potassium channel tetramerization domain–containing 10–dependent (KCTD10-dependent) pathway. This KIF13B/KCTD10 axis reduced KLF4 protein levels, thereby inhibiting the proinflammatory responses and fibroblast-like transition of VSMCs to preserve their contractile phenotype. Importantly, the adverse effects of *Kif13b* deficiency on atherogenesis were effectively rescued by the small-molecule KLF4 inhibitor Kenpaullone. Our results unveil a VSMC-specific atheroprotective role for KIF13B, define the KIF13B/KCTD10/KLF4 pathway as a key regulatory axis governing VSMC fate and plaque stability, and validate the therapeutic potential of KIF13B for treating advanced atherosclerosis.

## Abstract 2

<strong>Vascular Smooth Muscle Cell-derived KIF13B Protects Against Atherosclerosis: Evidence from Humans and Mice</strong>

## Graphical abstract

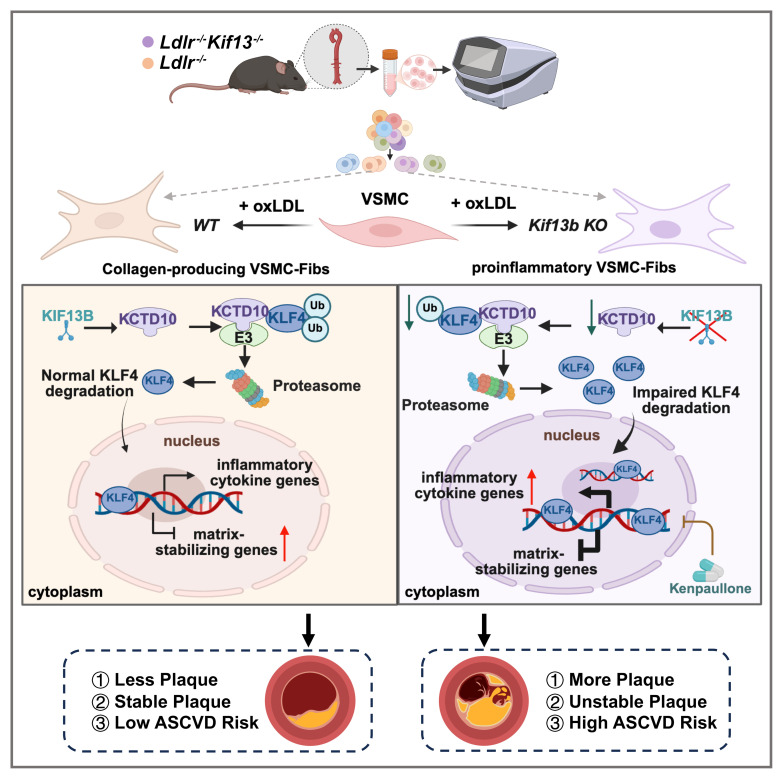

## Introduction

Atherosclerotic cardiovascular disease (ASCVD) continues to be the principal cause of global morbidity and mortality, creating a substantial economic burden. Notably, the majority of sudden deaths of patients with ASCVD are attributable to the rupture of unstable plaques and subsequent thrombotic events (1, 2). Current therapeutic strategies, predominantly centered on lipid-lowering (3), antiinflammation (4, 5), and antiplatelet therapies (6), have significantly improved clinical outcomes. Despite these advances, a residual cardiovascular risk persists (7), underscoring the urgent need to elucidate new pathogenic mechanisms and identify therapeutic targets for combating this pervasive disease.

The pathogenesis of atherosclerosis is driven by the interplay of multiple cell types, encompassing endothelial dysfunction, macrophage activation, and phenotypic switching of vascular smooth muscle cells (VSMCs), among which VSMCs emerge as central, multifaceted regulators of atherosclerotic plaque progression and stability (8). During the course of atherogenesis, VSMCs undergo a remarkable phenotypic transition characterized by dedifferentiation, migration, and transdifferentiation into diverse cell types, such as fibroblast-like, macrophage-like, foam cell, mesenchymal stem cell–like, myofibroblast-like, and osteochondral cell–like phenotypes (9–12). These switched VSMCs contribute to neointima formation, inflammation, vascular calcification, and secretion of extracellular matrix components essential for fibrous cap integrity, highlighting the dual nature of VSMCs in atherosclerosis. On the one hand, these cells are crucial for maintaining plaque stability; on the other hand, their dysfunction can actively trigger disease progression. Notwithstanding its pathophysiological implications, the precise molecular mechanisms governing VSMC phenotypic switching remain incompletely defined. Moreover, no therapeutic strategy has been approved by the FDA to delay atherosclerosis or stabilize plaques by specifically modulating VSMC phenotypic fate.

Recently, numerous studies have established a proatherogenic role for Krüppel-like factor 4 (KLF4), a zinc finger transcription factor regulating cell fate in VSMCs, where it drives phenotypic switching and represses the expression of key contractile markers, including *Acta2*, *Tagln*, *Myh11*, and *Cnn1* (13–17). Consistent with its atherosclerosis-prone role, VSMC-specific deletion of *Klf4* in murine models results in lesions that are 50% smaller and exhibit features of increased plaque stability, such as a 2-fold increase in the Acta2^+^ fibrous cap with a more than 60% decrease in VSMC-derived Lgals3^+^ cells (16). Thus, the KLF4-mediated phenotypic switching in VSMCs is a pivotal driver of atherosclerosis and represents a highly promising therapeutic target. Nevertheless, clinically applicable interventions targeting KLF4 are currently unavailable, and their therapeutic potential remains unrealized yet.

Kinesin family member 13B (KIF13B) is a motor protein primarily implicated in intracellular trafficking (18, 19). Emerging evidence has demonstrated a fundamental role of KIF13B in lipid metabolism and vascular disease (20, 21). Our previous study showed that macrophage-specific KIF13B prevents atherosclerosis by maintaining efficient macrophage efferocytosis (22). However, given that VSMCs and their phenotypically modulated derivatives constitute the majority of cells within advanced human atherosclerotic plaques, the function of KIF13B within this critical cell lineage is entirely unknown, and the specific effect of VSMC-derived KIF13B on atherosclerotic plaque development and stability has not been investigated to our knowledge.

In this study, we examined the role of KIF13B in VSMCs and its potential as a therapeutic target in atherosclerosis. We observed that VSMC-specific deficiency of *Kif13b* aggravated atherogenesis and impaired plaque stability. Mechanistically, KIF13B promoted KCTD10-dependent ubiquitination and degradation of KLF4, thereby suppressing the proinflammatory fibroblast-like transition of VSMCs and preserving a collagen-producing phenotype. Importantly, the pathological exacerbation due to KIF13B deficiency was effectively reversed by Kenpaullone, a small-molecule inhibitor of KLF4. Our findings unveil a VSMC-specific function of KIF13B and position the KIF13B/KCTD10/KLF4 pathway as a compelling target for stabilizing vulnerable plaques.

## Results

### Decreased expression of KIF13B in VSMCs is linked to the progression of atherosclerosis in humans.

**Figure 1:**
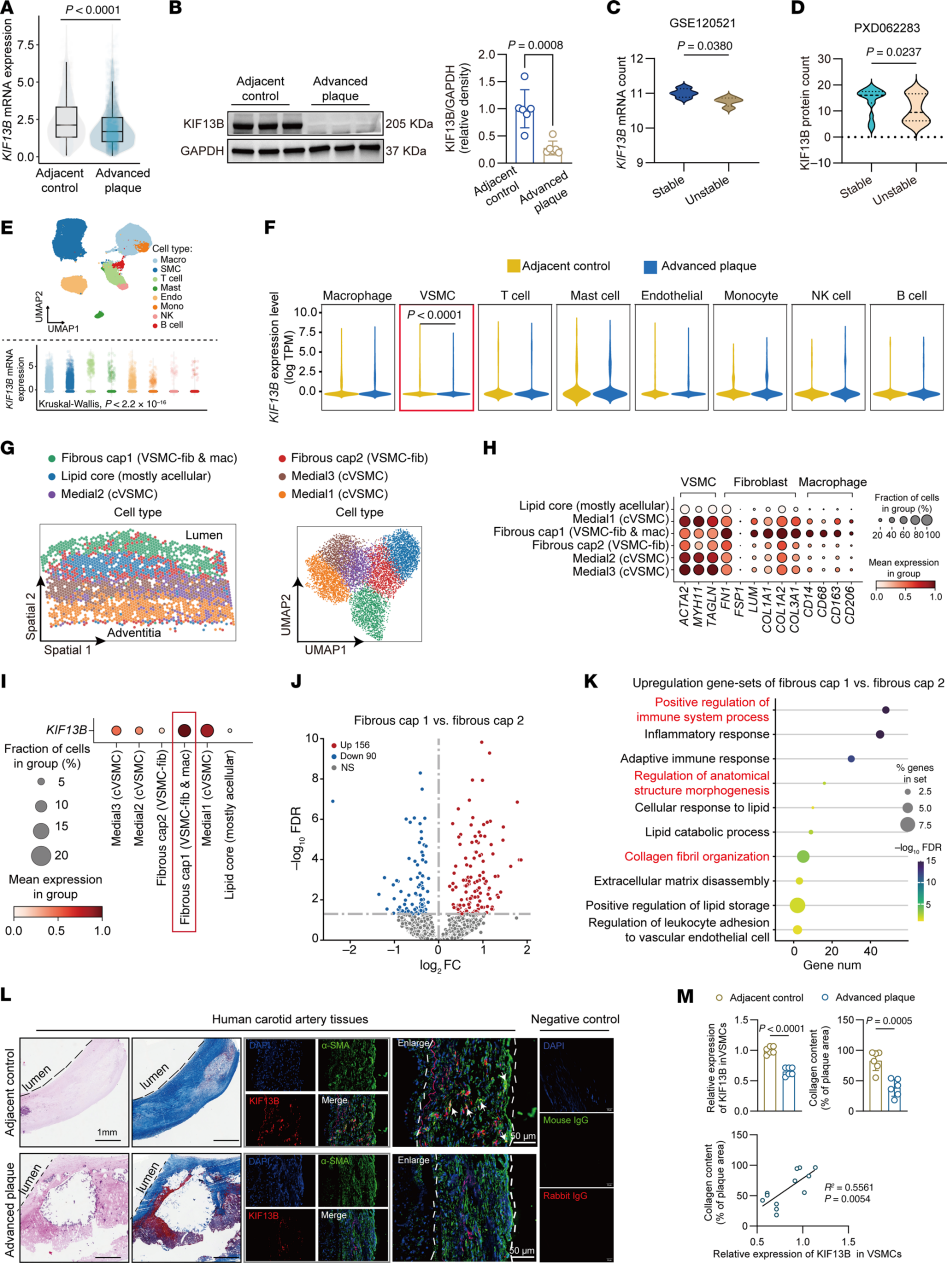
Decreased expression of KIF13B in VSMCs is associated with more severe human atherosclerosis. (**A**) scRNA-seq analysis of *KIF13B* mRNA levels in advanced plaques versus adjacent control tissues (*n* = 4 per group). (**B**) Western blotting for KIF13B protein expression in advanced plaques versus adjacent control tissues (*n* = 6 per group). (**C** and **D**) Violin plot of *KIF13B* mRNA (**C**) and KIF13B protein (**D**) expression in stable versus unstable human plaques from GEO GSE120521 (*n* = 4 per group) and ProteomeXchange PXD062283 (stable *n* = 19, unstable *n* = 38), respectively. (**E**) Uniform manifold approximation and projection (UMAP) visualization of scRNA-seq profiles in human atherosclerotic plaque tissues. Scatter plot shows cell-type scale of *KIF13B* mRNA expression among different cells in human atherosclerotic plaque tissues. (**F**) Violin plots of *KIF13B* mRNA expression among different cells in human atherosclerotic plaque tissues. (**G**) Left: Human plaque overlaid with capture spot cluster annotations. Right: UMAP of capture spots colored by human plaque-cluster affiliation. (**H**) Dot plot showing expression of cell-type markers in clusters. Dots are colored by minimum-maximum scaled gene expression level. (**I**) Dot plot showing *KIF13B* mRNA expression among the different cell clusters annotated in **G**. (**J**) Volcano plot of DEGs between fibrous cap 1 and cap 2 VSMCs, with upregulated genes in red, downregulated genes in blue, and nonsignificant genes in gray. (**K**) GO enrichment analysis of DEGs between fibrous cap 1 and cap 2 VSMCs. (**L**) Representative images of H&E staining, Masson’s staining, and immunofluorescence staining for α-SMA and KIF13B in human carotid artery plaque tissues (*n* = 6 per group). Scale bars: 1 mm and 50 μm; original magnification, ×2.09. (**M**) Quantitative analysis of KIF13B and α-SMA colocalization and collagen content and their correlation. The lesion areas are outlined by white dashed lines, and the white arrows indicate the positive staining of KIF13B in smooth muscle cells. Data are presented as the mean ± SEM and were analyzed by 2-tailed, unpaired Student’s *t* test (**B**–**D**, and **M**) or the Wilcoxon rank-sum test (**A** and **F**).

To investigate the potential role of KIF13B in atherogenesis, we first measured KIF13B mRNA and protein levels in advanced human aortic plaques and adjacent control tissues. We observed a significant reduction in KIF13B expression in advanced plaques (Figure 1, A and B). We further examined KIF13B expression in stable versus unstable plaques using datasets from the Gene Expression Omnibus (GEO) database. Consistent with our initial findings, both mRNA and protein levels of KIF13B were significantly lower in unstable plaques (Figure 1, C and D). Recent independent research has investigated the involvement of DNA methylation in the etiopathogenesis of atherosclerosis, documenting the role of this mechanism in various aspects of the disease (23, 24). However, it is unclear whether regulation of KIF13B expression by DNA methylation occurs in aortic plaque tissues. Next, we integrated 3 independent public databases regarding gene methylation. Our findings revealed that the methylation level of *KIF13B* was higher in atherosclerotic plaques than in healthy arterial tissues (Supplemental Figure 1, A and B; supplemental material available online with this article; https://doi.org/10.1172/JCI194175DS1). Even more remarkably, when compared with the asymptomatic atherosclerotic plaques, the methylation level of *KIF13B* was also significantly increased in the symptomatic atherosclerotic plaques (Supplemental Figure 1, C and D). Together, these results establish a negative correlation between KIF13B downregulation in plaques and the progression of human ASCVD.

Given that the stability of atherosclerotic plaques is governed by the combined actions of multiple cell types, to identify the cell types in which KIF13B undergoes the most substantial changes, we conducted single-cell RNA-seq (scRNA-seq) on human atherosclerotic plaque tissues. The data indicated widespread expression of *KIF13B* in various types of atherosclerotic plaque cells (Figure 1E). Nevertheless, it was only in VSMCs that the expression of *KIF13B* mRNA significantly diminished in unstable plaques (Figure 1F). Hence, we hypothesized that KIF13B in VSMCs may play a crucial role in the stability of atherosclerotic plaques. Subsequently, we analyzed aortic samples containing fibrous caps of early plaques through spatial transcriptomics (25) to explore the expression of KIF13B in VSMCs and their spatial distribution, with the aim of clarifying the effect of KIF13B on the functions of VSMCs. Through in-depth analysis, we discovered that VSMCs were distributed within the fibrous cap, lipid core, and media of atherosclerotic plaques (Figure 1G). These cells not only expressed traditional contractile markers but also the marker genes related to the characteristics of fibroblasts and macrophages (Figure 1H). We found that the fibrous cap contained 2 types of VSMC-derived cells, which we named fibrous cap 1 VSMCs and fibrous cap 2 VSMCs (Figure 1G). *KIF13B* was highly expressed in fibrous cap 1 VSMCs expressing contraction markers (*ACTA2*, *MYH11*, *TAGLN*), fibroblast markers (*FN1*, *LUM*, *COL1A2*), and M2 macrophage markers (*CD163*, *CD206*), while it was expressed at very low level sin fibrous cap 2 VSMCs (Figure 1, H and I). Gene ontology (GO) enrichment analysis of differentially expressed genes (DEGs) between fibrous cap1 and fibrous cap 2 VSMCs showed that the upregulated genes in fibrous cap 1 VSMCs with elevated expression of KIF13B were primarily enriched in biological processes, including positive regulation of the immune system process, regulation of anatomical structure morphogenesis, and collagen fibril organization (Figure 1, J and K). Furthermore, histopathological analysis indicated that the content of KIF13B protein in VSMCs and collagen content were decreased in advanced atherosclerotic plaques compared with adjacent control (Figure 1, L and M). Notably, KIF13B expression in VSMCs showed a positive correlation with collagen content within human atherosclerotic plaques (Figure 1M). The above results collectively demonstrate that the expression of KIF13B in VSMCs was closely related to the stability of human atherosclerotic plaques.

### Depletion of Kif13b in VSMCs exacerbates atherosclerosis in mice.

**Figure 2:**
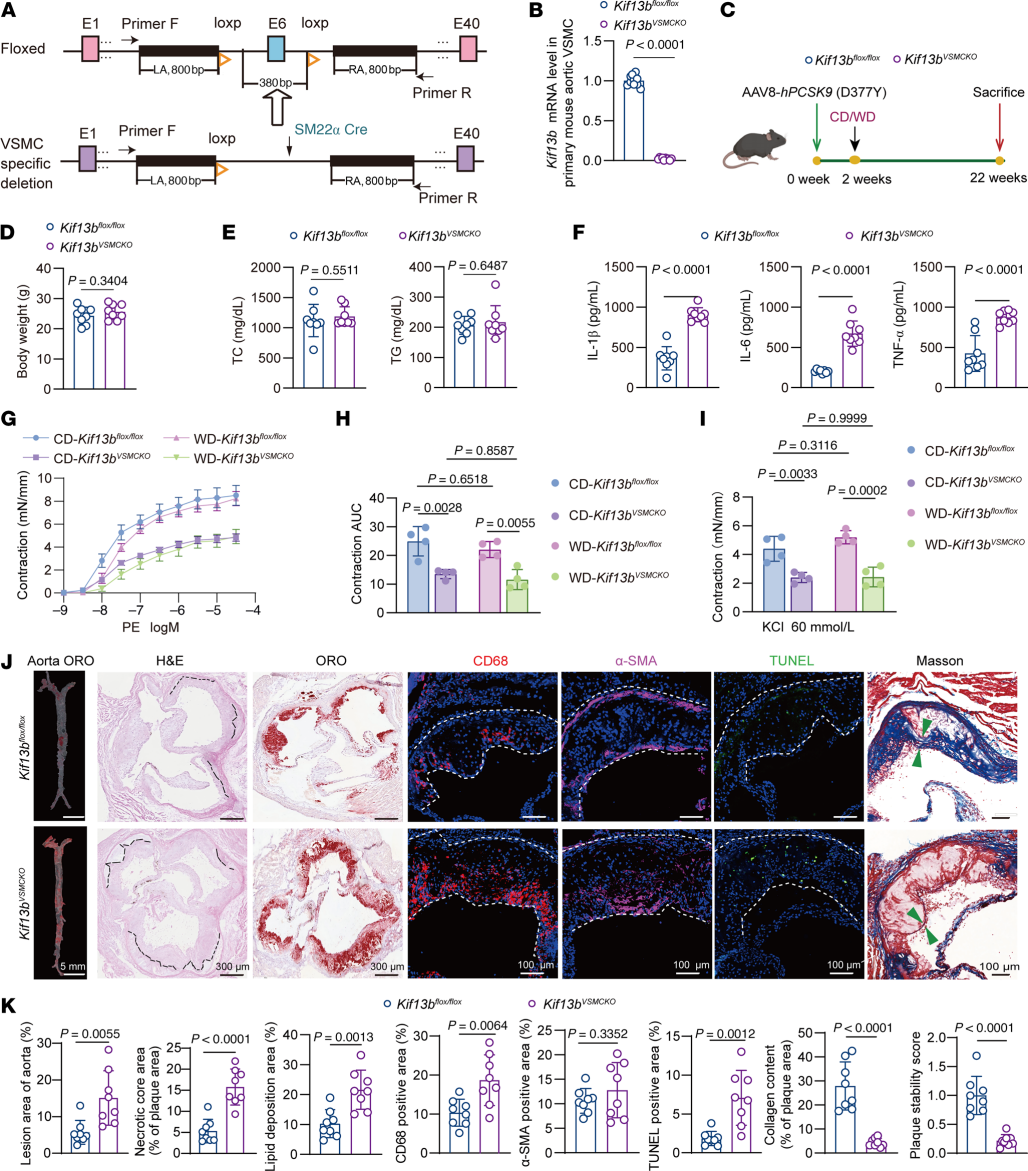
VSMC-specific *Kif13b* KO exacerbates atherosclerosis in mice. (**A**) Schematic of *Kif13b^VSMCKO^* mouse generation. (**B**) *Kif13b* mRNA levels in primary mouse VSMCs. (**C**) Schematic of the experimental timeline: 8-week-old male *Kif13b^fl/fl^* and *Kif13b^VSMCKO^* mice were injected with AAV8-PCSK9-D377Y and then fed a WD or CD for 20 weeks. (**D**–**F**) Body weight (**D**), plasma lipid levels (TC and TG) (**E**), and proinflammatory cytokines (IL-1β, IL-6, and TNF-α) (**F**) in mice fed a WD for 20 weeks (*n* = 8 per group). (**G**–**I**) Phenylephrine-induced vasoconstriction in the aortic ring from *Kif13b^fl/fl^* and *Kif13b^VSMCKO^* mice injected with AAV8-PCSK9-D377Y and then fed a WD or CD for 20 weeks (*n* = 4 per group). PE, phenylephrine. (**J**) Representative images of en face lesions in the whole aorta and representative images of H&E, Oil Red O (ORO), immunofluorescence staining (CD68, α-SMA, and TUNEL), and Masson’s trichrome staining of the aortic root. The necrotic core and total lesion area are outlined in black and white dashed lines, respectively; green arrowheads point to the fibrous cap (*n* = 8 per group). (**K**) Quantitative analyses of the data presented in **J**, including the percentage of lesion area in the entire aorta, the relative areas of the necrotic core, CD68^+^ area, α-SMA^+^ area, and TUNEL^+^ area within the aortic root sections, the lipid deposition area of the aortic root, the percentage of collagen content in the plaque area, and the plaque stability score. The lesion areas are outlined by white dashed lines, and the green arrowheads show the thickness of the fibrous cap in the atherosclerotic plaque. Data are presented as the mean ± SEM and were analyzed by 2-tailed, unpaired Student’s *t* test (**B**, **D**–**F**, and **K**) or 2-way ANOVA with Tukey post hoc test (**H** and **I**).

To validate the effect of VSMC-derived KIF13B on atherosclerotic development and plaque stability, we generated VSMC-specific *Kif13b*-KO (*Kif13b^VSMCKO^*) mice (Figure 2A) and then confirmed the successful KO of the *Kif13b* gene in primary VSMCs isolated from mouse aortic tissues (Figure 2B). Next, *Kif13b^VSMCKO^* mice and littermate control *Kif13b^fl/fl^* mice were injected via the tail vein with adeno-associated virus type 8 (AAV8) expressing PCSK9-D377Y and then fed a Western diet (WD) or a chow diet (CD) for 20 weeks (Figure 2C). Intriguingly, we found that, compared with *Kif13b^fl/fl^* mice, *Kif13b^VSMCKO^* mice had identical body weights and plasma total cholesterol (TC) and triglyceride (TG) levels, but showed increased plasma levels of the proinflammatory cytokines IL-1β, IL-6, and TNF-α (Figure 2, D–F), indicating that depletion of VSMC-derived KIF13B promoted systemic inflammation. We then assessed the effect of VSMC *Kif13b* deficiency on adrenergic receptor alpha 1–mediated vasoconstriction using phenylephrine in aortic rings isolated from *Kif13b^VSMCKO^* and *Kif13b^fl/fl^* mice. We found that loss of KIF13B in VSMCs significantly reduced the maximal contraction response of the aortic ring to phenylephrine, independent of diet interventions (Figure 2, G–I). These findings suggest that KIF13B derived from VSMCs had a remarkably protective effect on vascular contractile function in a plasma lipid–independent manner.

It is widely recognized that the attenuation of vascular contraction function can elicit atherogenesis (26). Therefore, we investigated the role of VSMC-derived KIF13B deficiency in atherosclerosis development. The results of our pathological analysis of atherosclerosis indicated an increase in atherosclerotic lesion areas in both the entire aorta and the aortic root, with larger necrotic core areas, enhanced CD68^+^ macrophage infiltration, and more TUNEL^+^ apoptotic cells in *Kif13b^VSMCKO^* mice; however, no difference in the α–smooth muscle actin (α-SMA) content was observed between the 2 groups (Figure 2, J and K). Moreover, Masson’s trichrome staining showed a significant reduction in collagen content in the fibrous cap of *Kif13b^VSMCKO^* mice (Figure 2, J and K), leading to plaque instability.

### Loss of KIF13B in VSMC does not affect intestinal morphology or lipid absorption.

**Figure 3:**
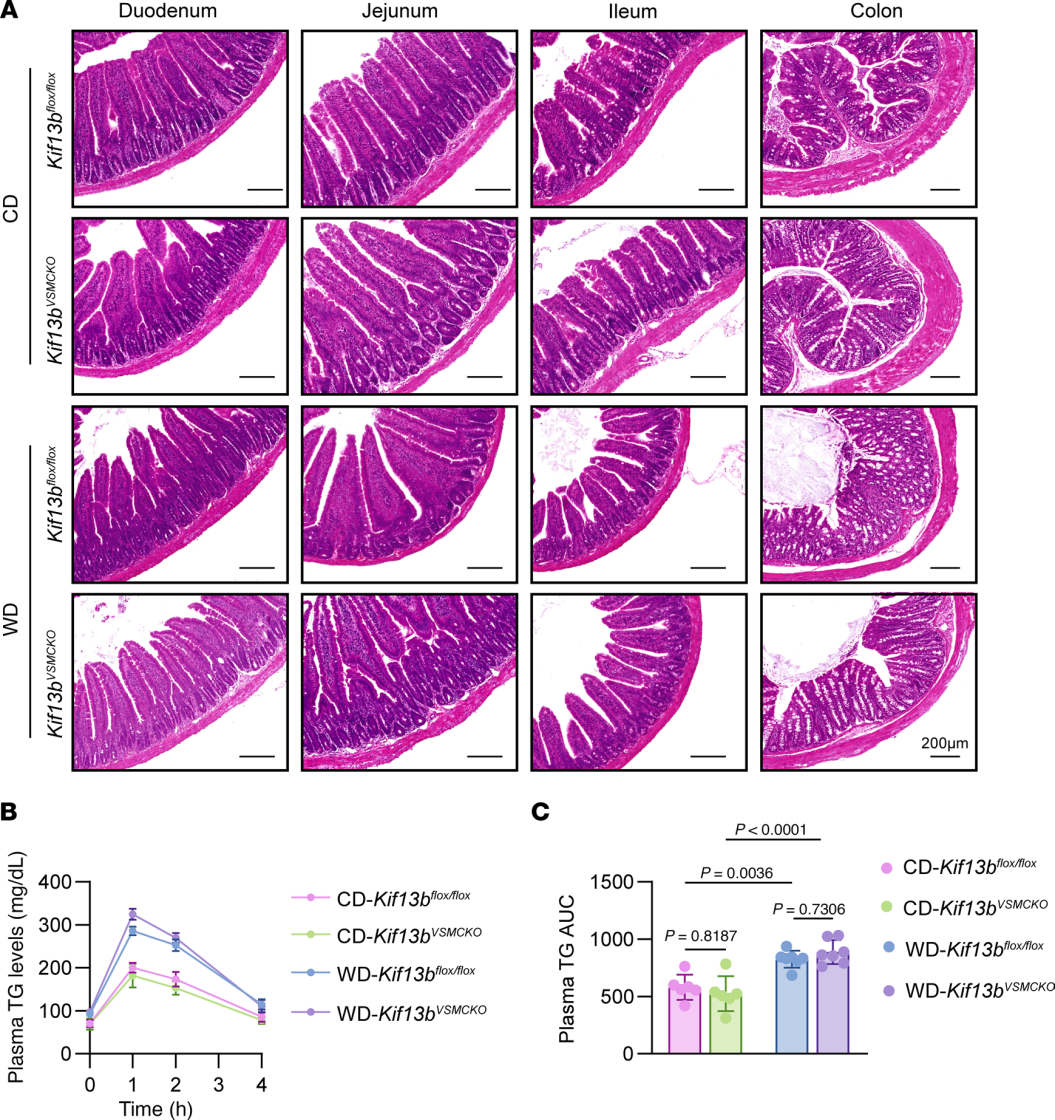
VSMC-specific *Kif13b* deletion does not affect intestinal morphology or lipid absorption. Eight-week-old male *Kif13b^fl/fl^* and *Kif13b^VSMCKO^* mice were injected with AAV8-PCSK9-D377Y for 2 weeks and then fed a WD (*n* = 7 per group) or CD (*n* = 6 per group) for 20 weeks. (**A**) Representative images of H&E staining of intestinal segments (duodenum, jejunum, ileum, colon) show no morphological differences between the 2 genotypes. Scale bars: 200 μm. (**B**) Oral lipid tolerance test. Mice were fasted for 4 hours, followed by oral gavage of olive oil (10 μL/g), and then plasma TG levels were measured at each subsequent time point. (**C**) Quantitative analyses of the AUC in **B**. Data are presented as the mean ± SEM and were analyzed by 2-way ANOVA with Tukey post hoc test (**C**).

Since VSMCs, as the principal contractile elements in blood vessels and hollow organs, such as the intestine and urinary bladder, have essential physiological functions in these systems, and TAGLN-Cre has been reported to delete floxed genes in the intestine and bladder (27, 28), we performed a histological examination of multiple intestinal segments. Our analysis revealed no significant morphological differences in the duodenum, jejunum, ileum, or large intestine between *Kif13b^fl/fl^* and *Kif13b^VSMCKO^* mice under either hypercholesterolemic or normocholesterolemic conditions (Figure 3A). To further evaluate whether VSMC-specific deletion of *Kif13b* affects intestinal lipid absorption, we measured TG levels in plasma from *Kif13b^fl/fl^* and *Kif13b^VSMCKO^* mice after an overnight fast followed by oral olive oil gavage. Results from this lipid tolerance test demonstrated that *Kif13b* deficiency in VSMCs did not alter circulating TG concentrations under either dietary condition (Figure 3, B and C), indicating that *Kif13b^VSMCKO^* mice had normal intestinal morphology and intact intestinal function.

### Depletion of KIF13B in VSMC does not induce maladaptive bladder remodeling resembling megabladder.

**Figure 4:**
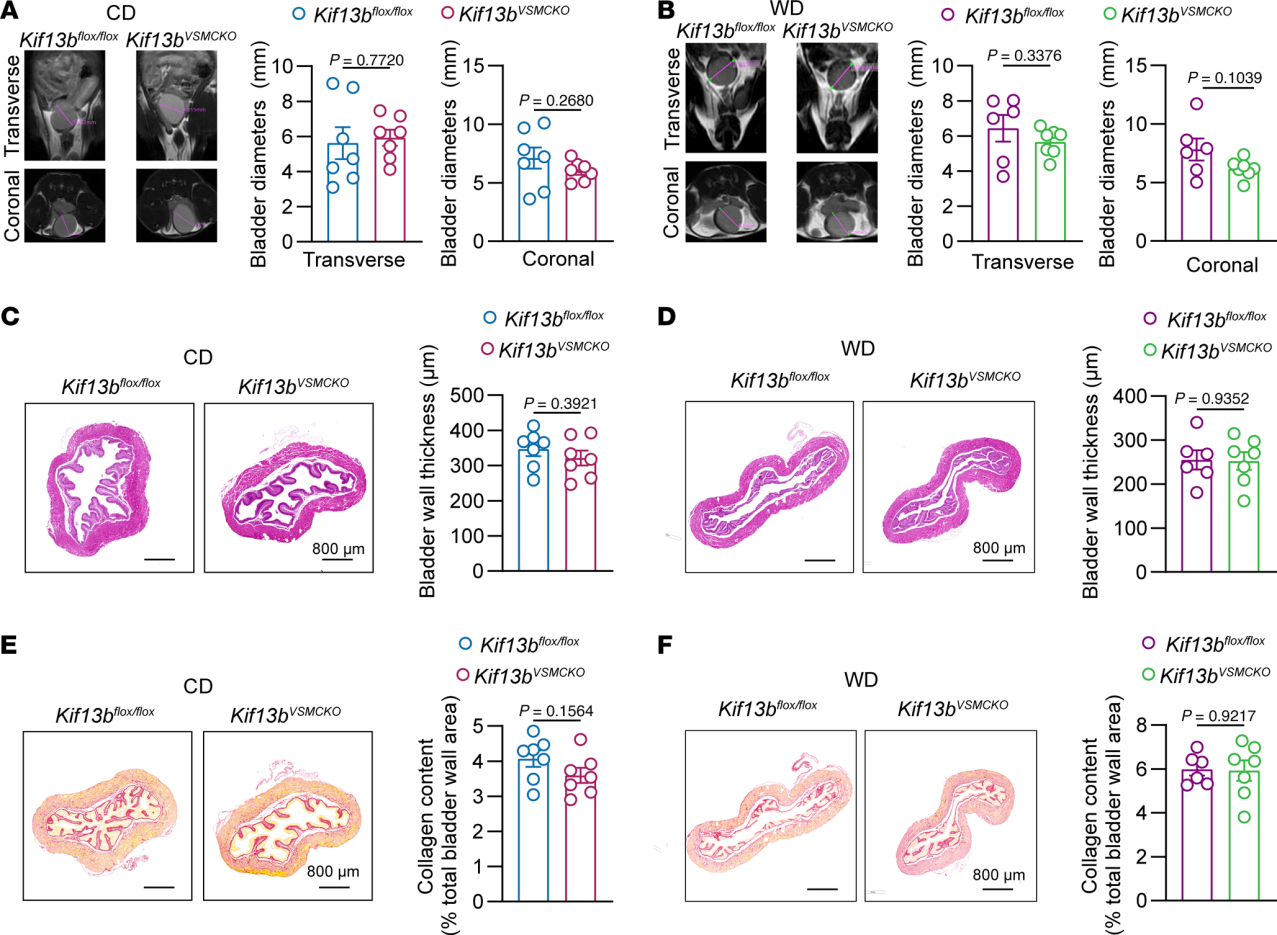
*Kif13b* deficiency in VSMCs does not cause bladder remodeling. Eight-week-old male *Kif13b^fl/fl^* and *Kif13b^VSMCKO^* mice were injected with AAV8-PCSK9-D377Y for 2 weeks and then fed a WD or CD for 20 weeks (*n* = 7 per group). (**A** and **B**) Representative images and quantitative analysis of bladder volume shown by MRI of *Kif13b^fl/fl^* and *Kif13b^VSMCKO^* mice. (**C** and **D**) Representative images of H&E staining and quantitative analysis of bladder wall thickness in *Kif13b^fl/fl^* and *Kif13b^VSMCKO^* mice. Scale bars: 800 μm. (**E** and **F**) Representative images of Picrosirius red staining and quantitative analysis of collagen deposition in *Kif13b^fl/fl^* and *Kif13b^VSMCKO^* mice. Scale bars: 800 μm. Data are presented as the mean ± SEM and were analyzed by 2-tailed, unpaired Student’s *t* test (**A**–**F**).

Previous studies have also reported that the decline in VSMC contractile function caused by VSMC phenotypic remodeling can lead to maladaptive bladder remodeling resembling megabladder in humans and mice (27–29). To determine whether VSMC-restricted KIF13B deficiency has any effects on bladder function, we examined bladder morphology using MRI of *Kif13b^fl/fl^* and *Kif13b^VSMCKO^* mice under CD and WD conditions. Strikingly, we found that VSMC-specific *Kif13b* deficiency had urinary bladder volume identical to that of *Kif13b^fl/fl^* mice fed a CD and WD (Figure 4, A and B). Further characterization of the grossly dilated bladder revealed similarities in bladder wall thickness and collagen deposition in both *Kif13b^fl/fl^* and *Kif13b^VSMCKO^* mice (Figure 4, C–F). Collectively, these data show that KIF13B in VSMCs had no influence on maintaining the structural integrity of the bladder under hypercholesterolemic and normocholesterolemic conditions.

### Inhibiting KIF13B promotes proinflammatory fibroblast–like VSMC transition.

**Figure 5:**
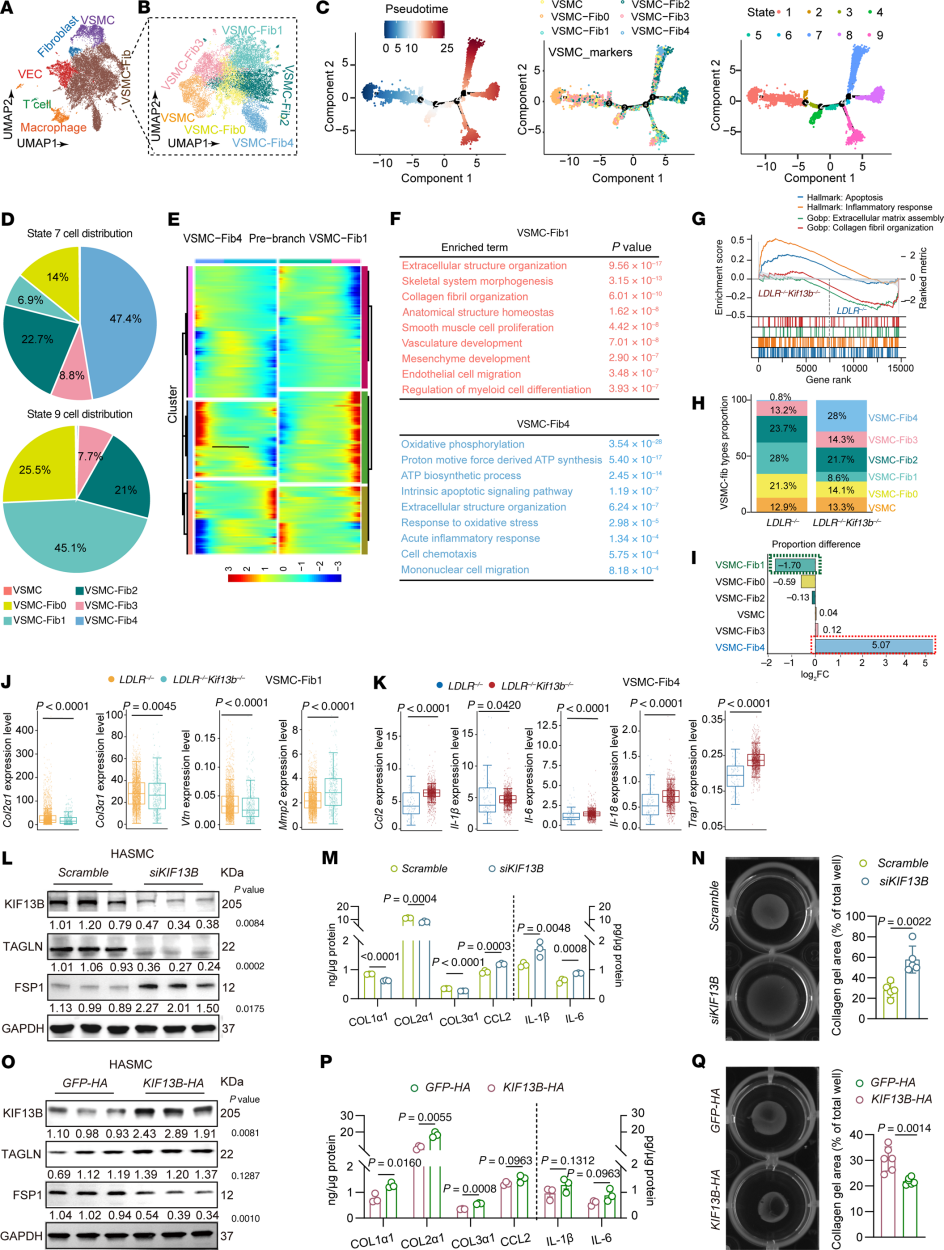
KIF13B directs VSMC transition to a matrix-stabilizing phenotype. (**A**) UMAP visualization of aorta scRNA-seq samples in *Ldlr^–/–^* and *Ldlr^–/–^ Kif13b^–/–^* mice fed a WD for 20 weeks (mix *n* = 5 into 1 sample). (**B**) UMAP visualization of VSMC-Fib subclustering analysis derived from **A**. (**C**) Monocle analysis derived from **A**, showing the ordering of VSMC-Fibs along pseudotime marked by the VSMC-Fibs state. (**D**) Distribution of VSMC-Fibs from different cell states based on the data in **C**. (**E**) Heatmap of different blocks of DEGs along the pseudotime trajectory. (**F**) Selected top GO terms related to the corresponding DEGs in **E**. (**G**) GSEA of VSMC-Fib aorta scRNA-seq data for *Ldlr^–/–^* and *Ldlr^–/–^ Kif13b^–/–^* mice, derived from A. (**H**) Stacked bar plot showing the proportion of aortic cells in *Ldlr^–/–^* and *Ldlr^–/–^ Kif13b^–/–^* mice from scRNA-seq data derived from **A**. (**I**) Bar plot showing the difference in cell proportions (log_2_ fold change [FC]) between *Ldlr^–/–^* and *Ldlr^–/–^ Kif13b^–/–^* mice for aorta scRNA-seq data derived from **A**. (**J**) Expression of *Col2a1*, *Col3a1*, *Vnt*, and *Mmp2* mRNA in VSMC-Fib1 cells from the scRNA-seq data on aortic cells from *Ldlr^–/–^* and *Ldlr^–/–^ Kif13b^–/–^* mice from **A**. (**K**) Expression of *Ccl2*, *Il1b*, *Il6*, *Il18*, and *Trap1* mRN in VSMC-Fib1 cells from the scRNA-seq data of aortic cells from *Ldlr^–/–^* and *Ldlr^–/–^ Kif13b^–/–^* mice from **A**. (**L**) Representative Western blot images and quantitative analysis of KIF13B, TAGLN, and fibroblast-specific protein 1 (FSP1) protein expression in HASMCs transfected with scramble or *siKIF13B* and subsequently treated with oxidized low-density lipoprotein (oxLDL) (50 μg/mL) for 48 hours (*n* = 3 independent biological replicates). (**M**) ELISA of supernatant COL1α1, COL2α1, COL3α1, CCL2, IL-1β, and IL-6 in HASMCs transfected with scramble or *siKIF13B* and subsequently treated with oxLDL (50 μg/mL) for 48 hours (*n* = 3 independent biological replicates). (**N**) HASMCs were transfected with scramble or *siKIF13B* and subsequently treated with oxLDL (50 μg/mL) for 48 hours, mixed with ice-cold collagen at a 1:4 ratio, and allowed to polymerize for 24 hours. Collagen gel size was subsequently measured (*n* = 5 independent biological replicates). (**O**) Representative Western blots and quantitative analysis of KIF13B, TAGLN, and FSP1 protein expression in HASMCs infected with lentivirus (LV) expressing *GFP-HA* or *KIF13B-HA* and subsequently treated with oxLDL (50 μg/mL) for 48 hours (*n* = 3 independent biological replicates). (**P**) ELISA of supernatant COL1α1, COL2α1, COL3α1, CCL2, IL-1β, and IL-6 content in HASMCs infected with LV expressing *GFP-HA* or *KIF13B-HA* and subsequently treated with oxLDL (50 μg/mL) for 48 hours (*n* = 3 independent biological replicates). (**Q**) HASMCs were infected with LV expressing *GFP-HA* or *KIF13B-HA* and subsequently treated with oxLDL (50 μg/mL) for 48 hours, mixed with ice-cold collagen at a 1:4 ratio, and allowed to polymerize for 24 hours. Collagen gel size was subsequently measured (*n* = 6 independent biological replicates). Data are presented as the mean ± SEM and were analyzed by Wilcoxon rank-sum test (**J** and **K**), 2-tailed, unpaired Student’s *t* test (**L**, **N**, **O**, and **Q**), or multiple unpaired *t* tests (**M** and **P**)**.**

To elucidate the characteristics of atherosclerotic plaques in the setting of KIF13B deficiency, we conducted in vivo scRNA-seq on *Ldlr^–/–^* and *Ldlr^–/–^ Kif13b^–/–^* mice after 20 weeks on a WD, which revealed that KIF13B inactivation markedly decreased collagen content in plaques with thinner fibrous caps (Supplemental Figure 2). Furthermore, our scRNA-seq data indicated that VSMC-derived fibroblast-like cells (VSMC-Fibs) were the predominant cell type in advanced-stage plaques (Figure 5A). Leiden clustering analysis further delineated that VSMCs could switch into 5 subtypes of VSMC-Fibs in advanced stages of mouse atherosclerosis (Figure 5B and Supplemental Figure 3A). Subsequently, we reconstructed a pseudotime trajectory aligned with VSMCs and VSMC-Fibs to model their phenotypic transition. These cells were categorized into 9 distinct states (Figure 5C). Among them, VSMC-Fib1 mapped to state 9, which was strongly associated with extracellular matrix stability, collagen fibril secretion, and vascular structural homeostasis, collectively conferring plaque-stabilizing characteristics. In contrast, VSMC-Fib4 localized to state 7 and exhibited a heightened proinflammatory response, indicative of a vulnerability-prone plaque phenotype (Figure 5, C–F). Gene set enrichment analysis (GSEA) further demonstrated that pathways related to apoptosis and inflammatory responses were significantly activated in *Kif13b*-deficient VSMC-Fibs, whereas extracellular matrix assembly and collagen fibril organization were markedly suppressed (Figure 5G). These findings demonstrate the observed vulnerable plaque phenotype in *Kif13b^VSMCKO^* mice. Consistent with this model, *Kif13b* deletion led to a marked reduction in VSMC-Fib1 cell numbers, alongside a notable increase in VSMC-Fib4 cells (Figures 5, H and I). To further validate these findings, we examined the VSMC-Fib1 cluster in *Ldlr^–/–^ Kif13b^–/–^* mice and observed significant downregulation of key stability-related genes (e.g., *Col2a1*, *Col3a1*, *Vnt*), along with a marked upregulation of the extracellular matrix-degrading protease *Mmp2* when compared with *Ldlr^–/–^* controls (Figure 5J). Conversely, the VSMC-Fib4 cluster from *Ldlr^–/–^ Kif13b^–/–^* mice showed significantly elevated expression of proinflammatory mediators, including *Ccl2*, *Il1b*, *Il6*, *Il18*, and *Trap1* (Figure 5K).

To directly assess the functional effect of KIF13B on VSMC phenotypic switching, we conducted complementary gain- and loss-of-function studies in primary human aortic smooth muscle cells (HASMCs). siRNA-mediated KIF13B knockdown promoted the phenotypic switch of HASMCs toward proinflammatory fibroblast-like cells (VSMC-Fibs), whereas KIF13B overexpression drove the transition toward a collagen-secreting, matrix-stabilizing phenotype (Figure 5, L and O). Consistent with these morphological changes, KIF13B knockdown significantly reduced collagen secretion and cell contractility, while enhancing the expression and secretion of inflammatory factors (Figure 5, M and N, and Supplemental Figure 3B). Conversely, KIF13B overexpression elevated collagen production and cell contractility (Figure 5, P and Q, and Supplemental Figure 3C). Taken together, our findings delineate an essential role of KIF13B in atherosclerotic plaque stabilization by directing VSMCs toward a matrix-preserving VSMC-Fib phenotype.

### KLF4 mediates KIF13B-dependent proinflammatory VSMC reprogramming.

**Figure 6:**
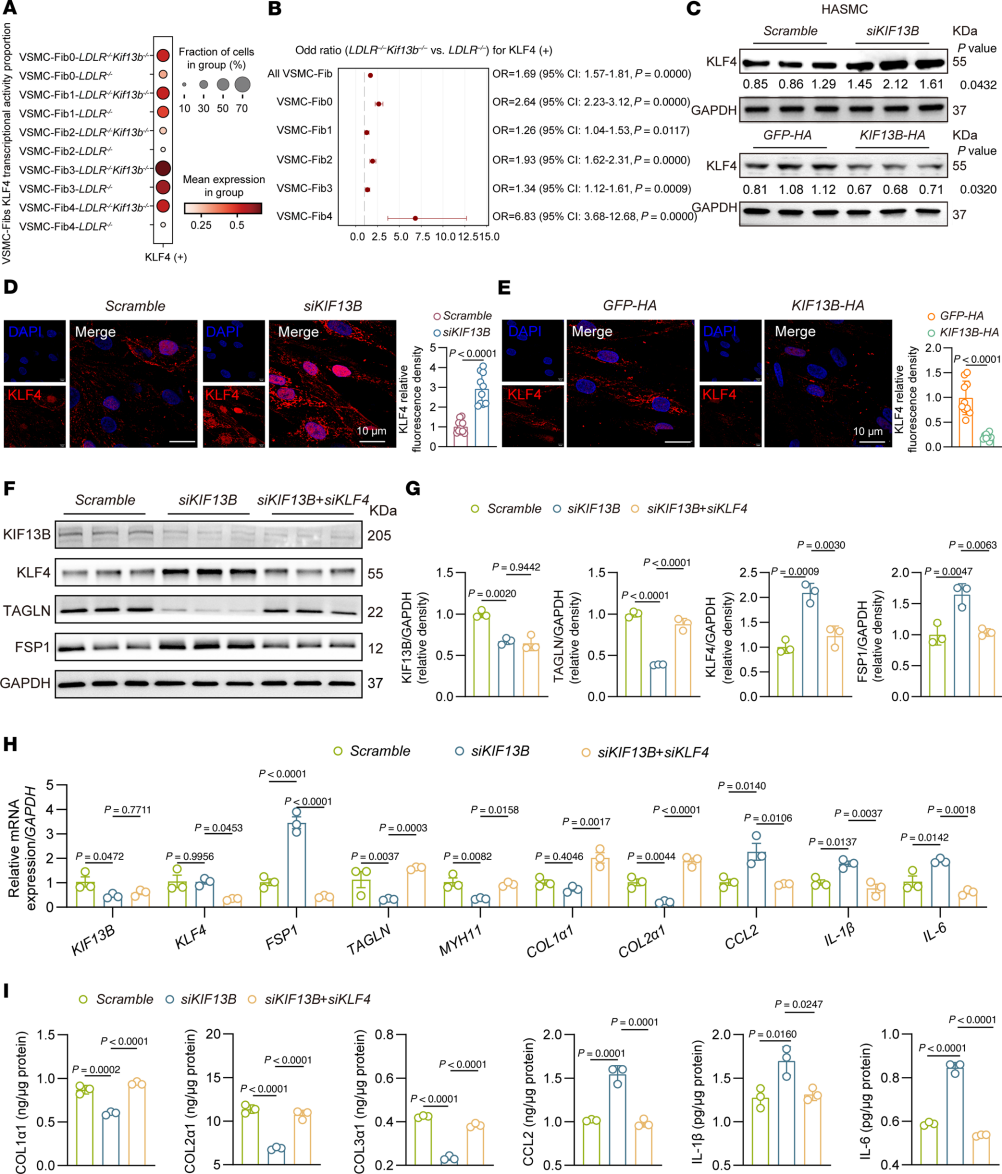
KLF4 acts downstream of KIF13B to drive proinflammatory reprogramming in VSMCs. (**A**) SCENIC analysis identified KLF4 as a key transcription factor activated in KIF13B-deficient VSMC-Fibs. (**B**) Visualization of forest plots of KLF4 transcriptional activity proportions in the VSMC-Fib subcluster of aorta scRNA-seq samples from *Ldlr^–/–^* and *Ldlr^–/–^ Kif13b^–/–^* mice fed a WD for 12 weeks. (**C**) Top: Representative Western blots and quantitative analysis of KLF4 protein expression in HASMCs transfected with scramble or *siKIF13B* and subsequently treated with oxLDL (50 μg/mL) for 48 hours (*n* = 3 independent biological replicate). Bottom: Representative Western blots and quantitative analysis of KLF4 protein expression in HASMCs infected with LV expressing *GFP-HA* or *KIF13B-HA* and subsequently treated with oxLDL (50 μg/mL) for 48 hours (*n* = 3 independent biological replicates). (**D** and **E**) Representative images and quantitative analyses of immunofluorescence staining for KLF4 and distribution in HASMCs transfected with scramble or *siKIF13B* (**D**) or infected with LV expressing *GFP-HA* or *KIF13B-HA* (**E**) for 48 hours (*n* = 10 independent biological replicates). Scale bars: 10 µm, original magnification, ×2.06. (**F** and **G**) Representative Western blots and quantitative analysis of KIF13B, KLF4, TAGLN, and FSP1 protein expression in HASMCs transfected with scramble, *siKIF13B*, or *siKIF13B* + *siKLF4* and subsequently treated with oxLDL (50 μg/mL) for 48 hours (*n* = 3 independent biological replicates). (**H**) Quantitative PCR analysis of VSMC-Fib phenotypic switching (*FSP1*, *TAGLN*, *MYH11*), collagen production (*COL1A1*, *COL2A1*, *COL3A1*), and inflammatory factor expression (*CCL2*, *IL1B*, *IL6*) in HASMCs transfected with scramble, *siKIF13B*, or *siKIF13B* +*s iKLF4* and subsequently treated with oxLDL (50 μg/mL) for 24 hours (*n* = 3 independent biological replicates). (**I**) ELISA detected supernatant COL1α1, COL2α1, COL3α1, CCL2, IL-1β, and IL-6 levels in HASMCs transfected with scramble or *siKIF13B* or *siKIF13B* + *siKLF4* and subsequently treated with oxLDL (50 μg/mL) for 48 hours (*n* = 3 independent biological replicates). Data are presented as the mean ± SEM and were analyzed by 2-tailed, unpaired Student’s *t* test (**C**–**E**) or 2-way ANOVA with Tukey post hoc test (**G**–**I**).

To explore the detailed molecular mechanism by which KIF13B governs the phenotypic transition of VSMCs, we performed a single-cell regulatory network inference and clustering (SCENIC) analysis of the DEGs in VSMC-Fibs from *Ldlr^–/–^* and *Ldlr^–/–^ Kif13b^–/–^* mice. The findings revealed that depletion of *Kif13b* in VSMCs significantly enhanced the transcriptional activity of KLF4, a key transcription factor for phenotypic switching in atherosclerosis and cancer (Figure 6, A and B). Western blot analysis demonstrated that knockdown of KIF13B in HASMCs upregulated the expression of KLF4, whereas overexpression of KIF13B-suppressed KLF4 expression (Figure 6C). Immunofluorescence staining further validated our observations, showing that KIF13B exerted an inverse regulatory effect on the levels of KLF4 in HASMCs (Figure 6, D and E).

Next, to determine whether KIF13B regulates the VSMC phenotypic transition through KLF4, we used a dual-knockdown strategy in HASMCs. Silencing KIF13B alone significantly reduced collagen production and increased the secretion of inflammatory factors, which were rescued by concomitant knockdown of KLF4 (Figure 6, F–I), demonstrating that KLF4 is a crucial downstream mediator of KIF13B signaling in VSMC phenotypic switching.

### KIF13B facilitates KCTD10-mediated ubiquitination and degradation of KLF4.

**Figure 7:**
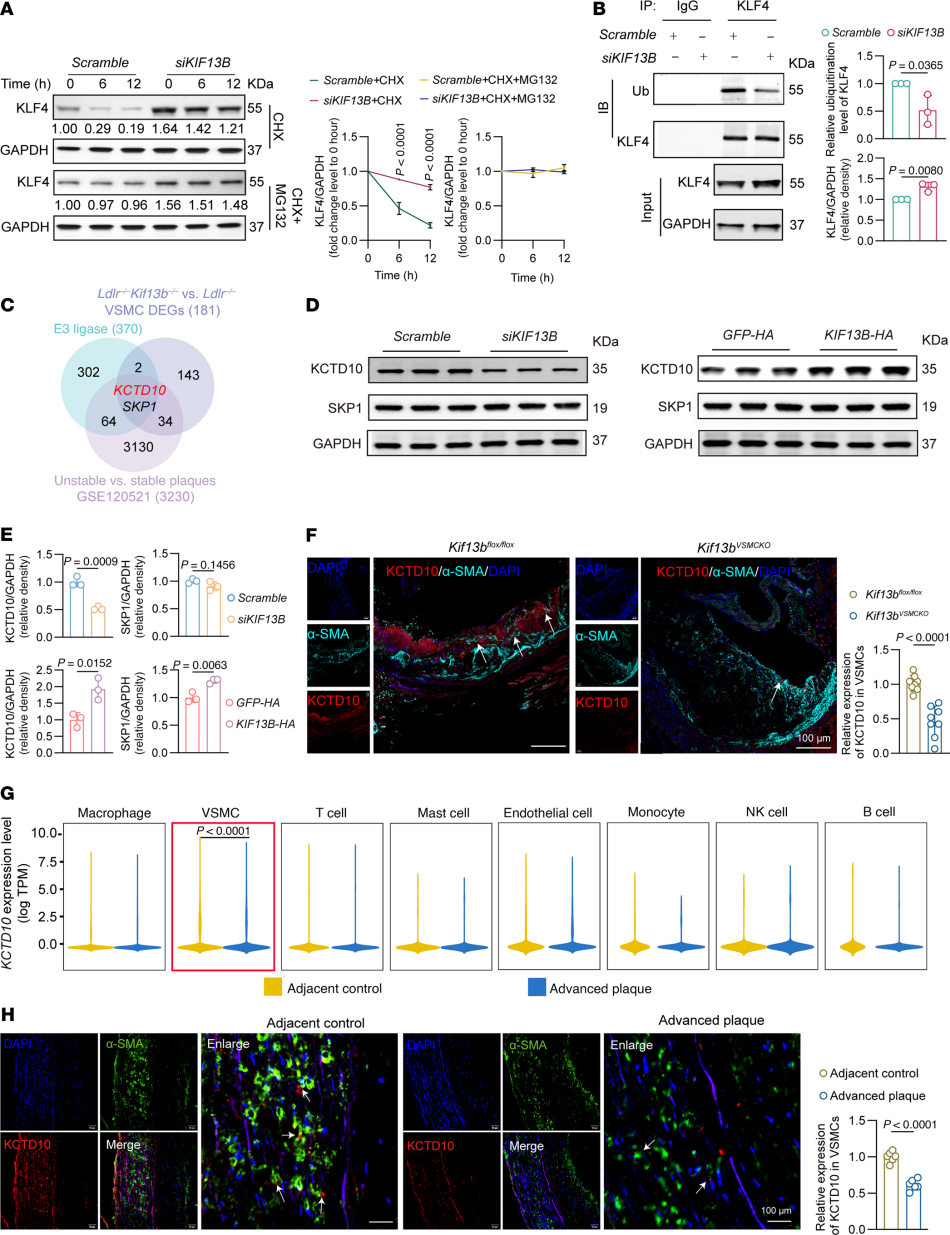
KIF13B drives KLF4 degradation in atherosclerosis through KCTD10-mediated ubiquitination. (**A**) Representative Western blots and quantitative analysis of KLF4 in HASMCs transfected with scramble or *siKIF13B* and treated with PBS or 10 μM MG132 in the presence of 50 μg/mL CHX for the indicated durations (*n* = 3 independent biological replicates). (**B**) Co-IP analysis and quantification of the ubiquitination of KLF4 (*n* = 3 independent biological replicates). (**C**) Venn diagram illustrates the intersection of commonly downregulated genes across DEGs in VSMCs from *Ldlr^–/–^ Kif13b^–/–^* versus *Ldlr^–/–^* mice fed a WD for 12 weeks, DEGs from unstable versus stable atherosclerotic plaques (GSE120521), and E3 ligase. DEGs with an FDR of less than 0.05 and a log_2_ FC of less than 0 were included. (**D**) Left: Representative Western blots of KCTD10 and SKP1 protein expression in HASMCs transfected with scramble or *siKIF13B* and subsequently treated with oxLDL (50 μg/mL) for 48 hours (*n* = 3 independent biological replicates). Right: Representative Western blots of KCTD10 and SKP1 protein expression in HASMCs infected with LV expressing *GFP-HA* or *KIF13B-HA* and subsequently treated with oxLDL (50 μg/mL) for 48 hours (*n* = 3 independent biological replicates). (**E**) Quantitative analysis of KCTD10 and SKP1 protein expression shown in **D**. (**F**) Immunofluorescence staining and quantification of α-SMA and KCTD10 in the aortic root of *Kif13b^fl/fl^* and *Kif13b^VSMCKO^* mice injected with AAV8-PCSK9-D377Y and then fed a WD for 20 weeks (*n* = 8 per group). (**G**) Violin plots of *KCTD10* mRNA expression in different cells in human atherosclerotic plaque tissues. (**H**) Immunofluorescence staining and quantification of α-SMA and KCTD10 expression in human carotid artery plaque tissues (*n* = 6 per group). White arrows indicate the content of KCTD10 in atherosclerotic plaque. Scale bars: 50 μm and 100 μm. Data are presented as the mean ± SEM and were analyzed by 2-way ANOVA with Tukey’s post hoc test (**A**), 2-tailed, unpaired Student’s *t* test (**B**, **F**, and **H**), or Wilcoxon rank-sum test (**G**).

Given that KIF13B does not directly regulate KLF4 transcription (Supplemental Figure 4A), we hypothesized that it may modulate KLF4 at the posttranslational level. To test this, we performed a cycloheximide (CHX) chase assay with or without the proteasome inhibitor MG132. Our results demonstrated that KIF13B knockdown stabilized the KLF4 protein by attenuating its proteasomal degradation (Figure 7A), which was further supported by co-IP experiments showing a reduction in KLF4 ubiquitination upon KIF13B knockdown (Figure 7B).

We next sought to identify the mechanism by which KIF13B regulates KLF4 ubiquitination. Integrated transcriptomics analysis of VSMCs from *Ldlr^–/–^ Kif13b^–/–^* versus *Ldlr^–/–^* mouse atherosclerotic tissues and of human unstable versus stable plaques revealed 2 downregulated ubiquitin ligase components: potassium channel tetramerization domain containing 10 (KCTD10) and S-phase kinase–associated protein 1 (SKP1) (Figure 7C). The Western blots showed that knockdown or overexpression of KIF13B specifically altered the expression of KCTD10, but not SKP1, in HASMCs (Figure 7, D and E). KCTD10 acts as a substrate receptor for really interesting new gene-type (RING-type) ubiquitin ligase complexes, and it has been implicated in cardiovascular disease (CVD) (30); however, its role in VSMCs and atherosclerosis remains unknown. We found that *Kif13b* deficiency also markedly reduced KCTD10 expression in VSMCs within mouse atherosclerotic plaques (Figure 7F). Analysis of human plaques further confirmed significantly reduced KCTD10 expression levels in VSMCs of advanced atherosclerotic plaques (Figure 7, G and H).

**Figure 8:**
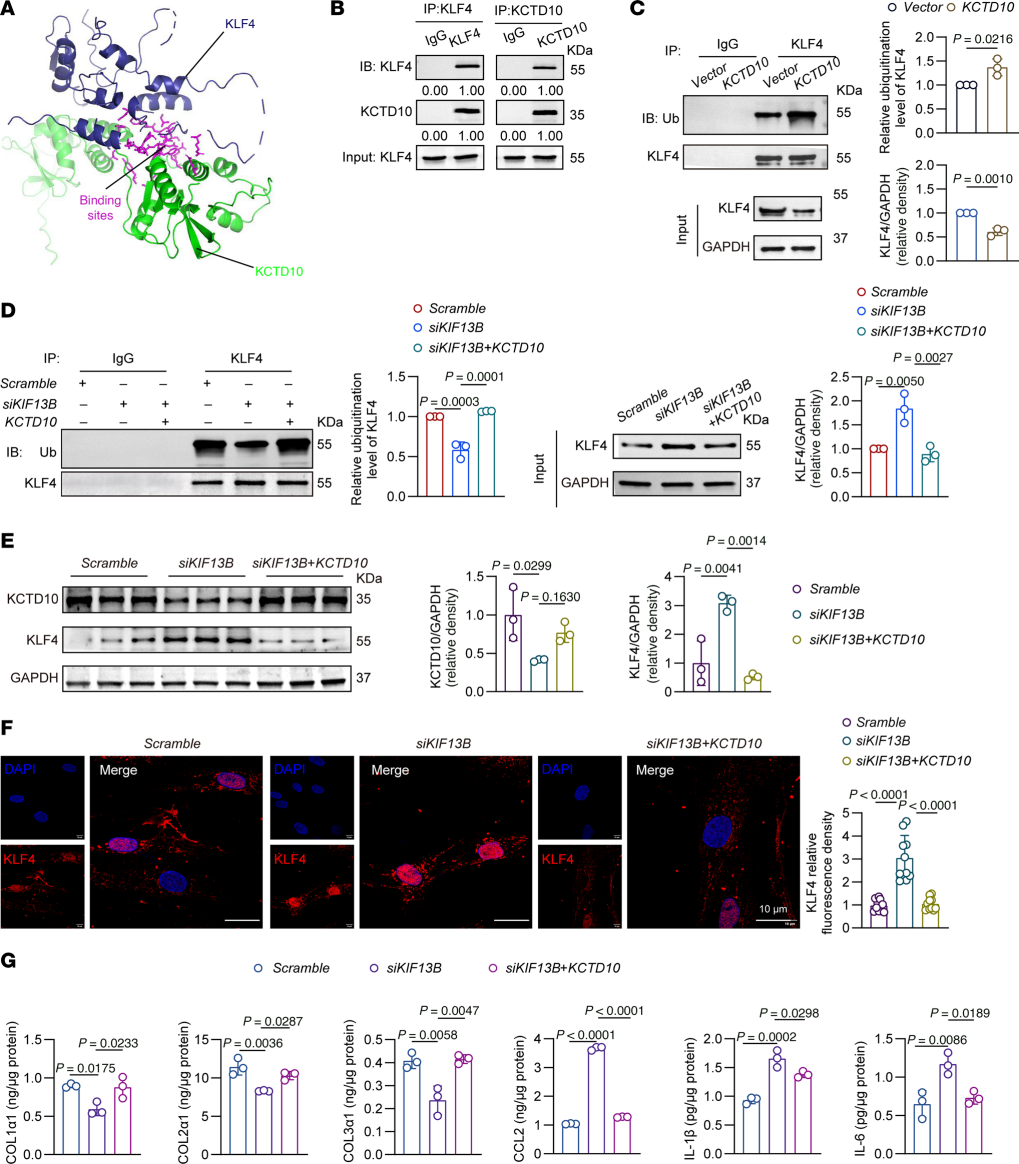
KIF13B promotes KLF4 ubiquitination and degradation via KCTD10. (**A**) Molecular docking prediction of KCTD10 and KLF4 interaction. (**B**) Quantitative analysis of co-IP assays confirmed the interaction between KCTD10 and KLF4 in HASMCs (*n* = 3 independent biological replicates). (**C**) Quantitative analysis of co-IP assays of the ubiquitination of KLF4 in HASMCs transfected with *KCTD10* plasmid for 48 hours (*n* = 3 independent biological replicates). (**D**) Quantitative analysis of co-IP assays of the ubiquitination of KLF4 in HASMCs transfected with scramble, *siKIF13B*, or *siKIF13B* + *KCTD10* for 48 hours (*n* = 3 independent biological replicates). (**E**) Representative Western blots and quantitative analysis of KLF4 in HASMCs transfected with scramble, *siKIF13B*, or *siKIF13B* + *KCTD10* and subsequently treated with oxLDL (50 μg/mL) for 48 hours (*n* = 3 independent biological replicates). (**F**) Representative images and quantitative analyses of immunofluorescence staining of KLF4 expression and distribution in HASMCs transfected with scramble, *siKIF13B*, or *siKIF13B* + *KCTD10* for 48 hours (*n* = 10 independent biological replicates). Scale bars: 10 µm; original magnification, ×2.06. (**G**) ELISA of supernatant COL1α1, COL2α1, COL3α1, CCL2, IL-1β, and IL-6 levels in HASMCs transfected with scramble, *siKIF13B*, or *siKIF13B* + *KCTD10* for 48 hours (*n* = 3 independent biological replicates). Data are presented as the mean ± SEM and were analyzed by 2-tailed, unpaired Student’s *t* test (**C**) or 1-way ANOVA with Tukey post hoc test (**D**–**G**).

To determine whether KIF13B regulates KLF4 stability through KCTD10, we first predicted a direct KCTD10-KLF4 interaction via molecular docking (Figure 8A). Co-IP assays then confirmed that KCTD10 binds to KLF4 and promotes its ubiquitination, thereby reducing KLF4 protein levels (Figure 8, B and C). Furthermore, the stabilization of KLF4 resulting from KIF13B knockdown was effectively reversed by KCTD10 overexpression (Figure 8D). Both Western blot and immunofluorescence analyses confirmed an inverse correlation between KCTD10 and KLF4 protein levels in HASMCs (Figure 8, E and F). Finally, the proinflammatory and collagen-suppressive phenotype induced by KIF13B knockdown was significantly rescued by KCTD10 overexpression (Figure 8G and Supplemental Figure 4B).

### Pharmacological inhibition of KLF4 with Kenpaullone alleviates atherosclerosis in mice.

**Figure 9:**
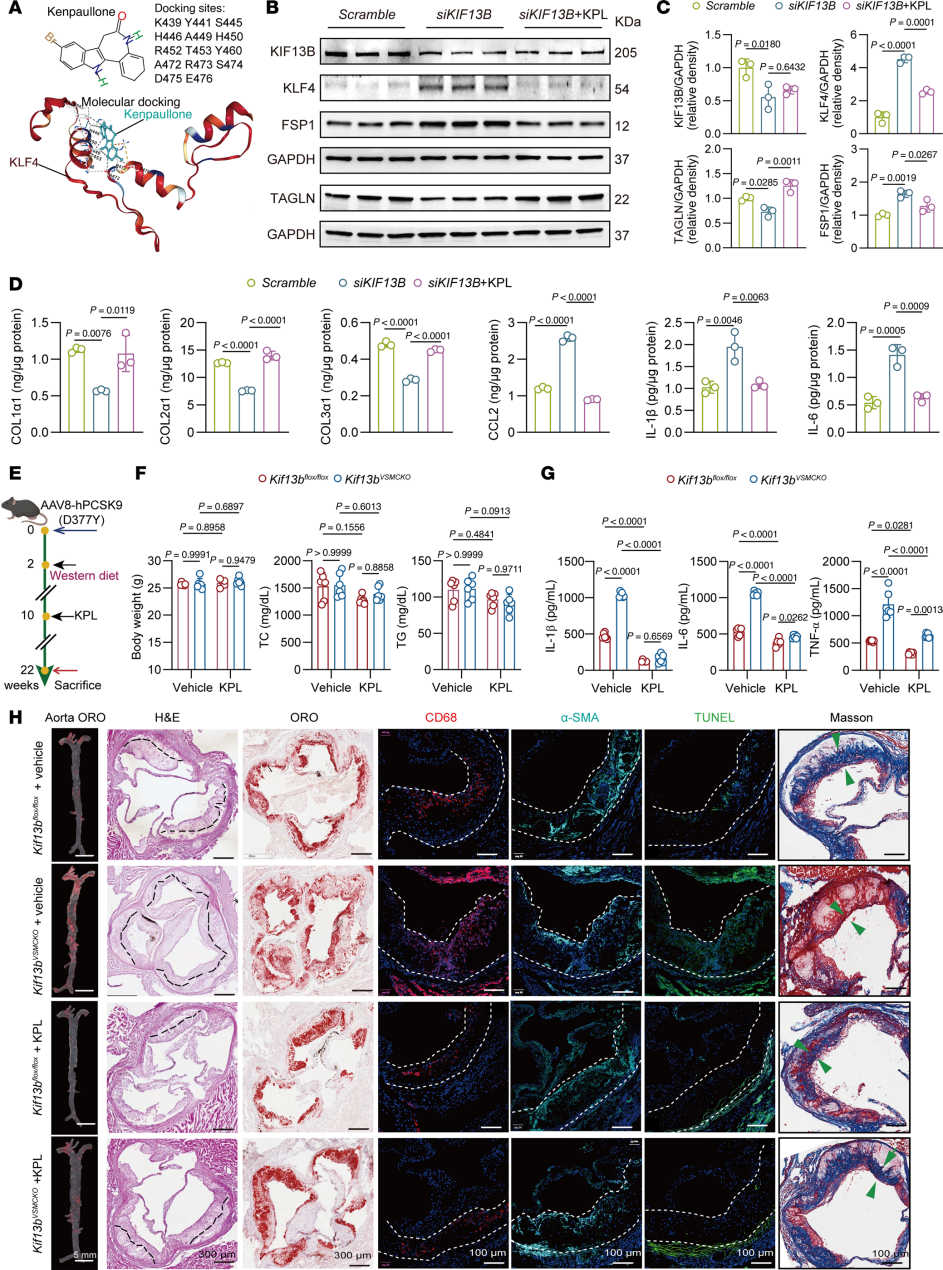
Pharmacological inhibition of KLF4 effectively ameliorates atherosclerosis in mice. (**A**) Schematic representation of the structure of Kenpaullone and its working model. (**B**) Representative Western blots of KIF13B, KLF4, FSP1, and TAGLN expression in HASMCs transfected with scramble, *siKIF13B*, or *siKIF13B* + Kenpaullone (10 μM) and subsequently treated with oxLDL (50 μg/mL) for 48 hours. (**C**) Quantitative analyses of the Western blotting in **B** (*n* = 3 independent biological replicates). (**D**) ELISA of supernatant COL1α1, COL2α1, COL3α1, CCL2, IL-1β, and IL-6 levels in HASMCs transfected with scramble, *siKIF13B*, or *siKIF13B* + Kenpaullone (10 μM) for 48 hours (*n* = 3 independent biological replicates). (**E**) Schematic of the experimental timeline: 8-week-old male *Kif13b^fl/fl^* and *Kif13b^VSMCKO^* mice received a single tail vein injection of AAV8-PCSK9-D377Y, followed 2 weeks later by feeding with a Western diet (WD) for another 20 weeks to induce advanced atherosclerosis. The mice received oral administration of Kenpaullone (1 mg/kg/d) for the last 12 weeks (n = 6 per group). (**F**) Body weights and plasma lipid levels (TC and TG) of the mice in **E**. (**G**) Proinflammatory cytokines (IL-1β, IL-6, TNF-α) of the mice in **E**. (**H**) Representative images of the en face lesions in the whole aorta and representative images of H&E staining, ORO staining, immunofluorescence staining (CD68, α-SMA, and TUNEL), and Masson’s trichrome staining of the aortic root. The necrotic core and total lesion area are outlined with black and white dashed lines, respectively; green arrowheads indicate the fibrous cap (*n* = 6 per group). Scale bars: 5 mm, 100 μm, and 300 μm. Data are presented as the mean ± SEM and were analyzed by 1-way ANOVA with Tukey post hoc test (**C** and **D**) or 2-way ANOVA with Tukey’s post hoc tests (**F** and **G**). KPL, Kenpaullone.

Finally, to explore a potential therapeutic strategy for atherosclerosis stemming from VSMC-specific *Kif13b* deficiency, we screened a small-molecule compound library and identified Kenpaullone as a KLF4 inhibitor (Figure 9A). Our reporter assays showed that Kenpaullone treatment significantly suppressed KLF4 promoter activity to reduce the mRNA expression level of *KLF4* (Supplemental Figure 5), suggesting that its downregulatory effect on KLF4 occurred at the transcriptional level. In HASMCs, Kenpaullone treatment effectively counteracted the effects of KIF13B knockdown by significantly reducing KLF4 protein levels and then enhancing collagen production and suppressing the secretion of inflammatory factors (Figure 9, B–D, and Supplemental Figure 6).

We next evaluated the in vivo therapeutic efficacy of Kenpaullone. Advanced atherosclerosis was induced in 8-week-old male *Kif13b^VSMCKO^* and *Kif13b^fl/fl^* mice injected with AAV8-PCSK9-D377Y via the tail vein and then fed a WD for 20 weeks, followed by oral administration of Kenpaullone (1 mg/kg/d) for the last 12 weeks (Figure 9E). Kenpaullone treatment had no obvious influence on body weight, plasma lipid profiles, or markers of liver toxicity, such as ALT and AST (Figure 9F and Supplemental Figure 7, A and B). However, it significantly reduced systemic inflammation, as evidenced by decreased plasma levels of IL-1β, IL-6, and TNF-α (Figure 9G). Histological analyses demonstrated that Kenpaullone attenuated the atherosclerotic lesion burden in both the aorta and aortic root, concomitant with a reduction in the necrotic core area, CD68^+^ macrophage infiltration, and TUNEL^+^ apoptotic cells, alongside an increase in collagen content (Figure 9H and Supplemental Figure 7C).

Collectively, these findings indicate that Kenpaullone impeded atherosclerosis progression by inhibiting KLF4 and the associated proinflammatory VSMC reprogramming without any detectable adverse effects, highlighting its potential as a promising therapeutic strategy.

## Discussion

Although VSMC phenotypic switching is central to plaque progression and fate, the molecular mechanisms that drive VSMCs toward maladaptive and proinflammatory states are incompletely understood. Here, we identify the motor protein KIF13B as a critical determinant of VSMC phenotypic commitment and plaque stability. Across human datasets and tissue analyses, KIF13B expression was reduced in unstable plaques, particularly in VSMCs. Using VSMC-specific *Kif13b* KO in mice, we show that loss of KIF13B aggravated atherosclerosis and compromised plaque stability independent of plasma lipids, with increased inflammation, apoptosis, and necrotic core area and reduced fibrous cap collagen. Mechanistically, KIF13B promoted the KCTD10-dependent ubiquitination and proteasomal degradation of KLF4, a master regulator of VSMC phenotypic switching. Disruption of this KIF13B/KCTD10/KLF4 axis elevated KLF4 protein, skewed VSMCs toward a proinflammatory fibroblast-like state, and then diminished matrix production, ultimately resulting in unstable plaque formation. Importantly, pharmacological inhibition of KLF4 with Kenpaullone effectively reversed these effects and improved plaque features in vivo, supporting the translational therapeutic potential by targeting this pathway.

Interestingly, we demonstrate a consistent downregulation of KIF13B expression at both mRNA and protein levels in symptomatic and histologically unstable human plaques, accompanied by increased KIF13B promoter methylation, which provides a compelling clinical association and human relevance. Single-cell and spatial transcriptomics further localized the most pronounced reduction of KIF13B to VSMCs within the fibrous cap. These convergent observations highlight a cell-type–specific link between reduced KIF13B and human plaque vulnerability. While the upstream drivers of KIF13B hypermethylation remain to be identified, these data position KIF13B as a dynamically regulated node at the interface of epigenetic remodeling, inflammation, and VSMC fate.

The main purpose of our study lies in moving beyond correlation to establish causality through cell-specific genetic manipulation. Our prior work has revealed that depletion of macrophage KIF13B protein in macrophages promotes atherosclerotic development (21, 22); however, we cannot exclude the possibility that VSMCs, the most abundant cell type in the vascular walls, participate in the pathogenesis of atherosclerosis. Therefore, the VSMC-specific *Kif13b*-KO mouse model (*Kif13b^VSMCKO^*) allowed us to isolate the role of VSMC-derived KIF13B from its potential functions in other vascular cell types (22). Consistently, despite unchanged body weight and plasma lipid profiles, *Kif13b^VSMCKO^* mice exhibited increased lesion burden and hallmarks of plaque instability with larger necrotic cores, more CD68^+^ macrophages and TUNEL^+^ cells, and reduced cap collagen. The concomitant impairment of phenylephrine-evoked vasoconstriction suggests that KIF13B contributed to VSMC contractile function, and its loss may have fostered low-shear, proinflammatory conditions that favored lesion growth and destabilization. Together, these results define a lipid-independent route to plaque vulnerability driven by maladaptive VSMC reprogramming.

Given the complex properties of VSMCs, lineage-tracing and transcriptomics technologies have been applied to investigate VSMC plasticity, giving rise to distinct phenotypes such as mesenchymal-like, fibroblast-like, and macrophage-like cells, a critical determinant of atherogenesis and plaque stability in both humans and mice (9, 31–33). In agreement with the previous findings indicating that VSMC phenotypic switching toward fibroblast-like cells could stabilize an atherosclerotic plaque (34), our high-resolution analysis revealed a KIF13B^hi^ subset of VSMC-Fib1, which was enriched in pathways for extracellular matrix organization and collagen fibrillogenesis programs associated with cap integrity, and a KIF13B^lo^ subset of VSMC-Fib4 marked by heightened inflammatory signaling. Deletion of KIF13B reduced VSMC-Fib1 and expanded VSMC-Fib4, consistent with bulk plaque phenotypes and transcriptional signatures showing downregulated expression of structural genes such as *Col2a1* and *Col3a1*, and upregulated expression of matrix-degrading and inflammatory mediator genes, including *Mmp2*, *Ccl2*, *Il1b*, and *Il6*. Complementary gain- and loss-of-function experiments in primary human VSMCs demonstrated that KIF13B promoted a collagen-producing program and restrained inflammatory cytokine secretion. Collectively, our scRNA-seq data not only confirmed the presence of a predominant VSMC-Fib population in advanced atherosclerotic plaques but also identified the motor protein KIF13B as a pivotal molecular switch governing the functional fate of these cells, thus delineating a distinct perspective on the regulation of VSMC phenotypic commitment and its implications for plaque pathology.

Recently, Pan and colleagues demonstrated that VSMC phenotypic switching in atherosclerosis shares profound similarities with tumor biology, encompassing genomic instability, cancer-like characteristics of VSMC-derived cells, activation of oncogenic regulatory networks, and a therapeutic response to anticancer agents targeting DNA repair (35). Building upon this paradigm and the recently established role of clonal hematopoiesis in atherogenesis (36–38), our findings provide direct mechanistic evidence that solidifies the concept of atherosclerosis as a VSMC-driven, tumor-like disorder that is pathologically defined by clonal expansion, profound phenotypic plasticity, and a dysregulated tissue microenvironment. This analogy is mechanistically solidified by our identification of KCTD10, a substrate adaptor of the Cul3-associated E3 ubiquitin ligase complex and tumor suppressor through promotion of oncoprotein degradation (39, 40), and KLF4 — two molecules with established roles in cancer — as integral components of the KIF13B pathway. Of note, unlike KLF4, which has been extensively studied in the field of vascular biology, a recent study implicated KCTD10 in CVD (30); however, the causal link between KCTD10 and atherosclerosis has not, to our knowledge, been documented. Here, our integrative analyses identify KCTD10 as a KIF13B-responsive E3 component. Our study also confirms KCTD10-KLF4 interaction and shows that KCTD10 promoted KLF4 ubiquitination and degradation. Overexpression of KCTD10 rescued the KIF13B-knockdown phenotype, restoring matrix programs and dampening inflammation. Functionally, KLF4 knockdown significantly reversed the effects of KIF13B loss on collagen and cytokines, placing KLF4 downstream of KIF13B/KCTD10 in VSMCs. Although how a kinesin motor promotes KCTD10 expression remains to be determined, possibilities include trafficking-dependent regulation of mRNA localization, protein stability, or signaling intermediates that control KCTD10 transcription.

It is well known that conventional lipid-lowering therapies are not applicable in the treatment of normolipidemic patients with ASCVD (41) because in this patient population, the primary risk source frequently stems from plaque vulnerability rather than the overall atherosclerotic burden caused by hyperlipidemia. In fact, accumulating clinical evidence suggests that residual ASCVD risk persists and continues to increase among patients, even when they are undergoing lipid-lowering treatment (42). Our findings suggest that this residual risk might be precisely driven by the “tumor-like” phenotypic dysregulation of VSMCs, a mechanism molecularly characterized in this study. Therefore, therapeutic strategies aimed directly at rectifying this cellular pathophysiology are urgently needed. Pharmacological KLF4 inhibition with Kenpaullone, a known anticancer agent (43), reduced systemic inflammatory cytokines, lessened lesion burden and necrotic core size, decreased macrophage infiltration and apoptosis, and increased fibrous cap collagen in vivo, without detectable effects on mouse body weight, plasma lipid levels, or hepatic injury markers. These results provide proof of concept that targeting the KIF13B/KCTD10/KLF4 axis can stabilize plaques by reprogramming the VSMC state, orthogonal to lipid lowering. Given the residual ASCVD risk in well-treated, often normolipidemic patients, therapies that directly enhance cap integrity and temper intraplaque inflammation are attractive complements to current standards of care. Kenpaullone itself may serve as a chemical starting point; however, next-generation KLF4 inhibitors with improved specificity and vascular targeting will likely be required for clinical translation. Furthermore, from a therapeutic perspective, while systemic delivery of KIF13B agonists may be challenging, the success of KLF4 inhibition offers a more readily tractable strategy. The lack of overt bladder or intestinal pathology in our *Kif13b^VSMCKO^* models is reassuring and suggests that targeting this pathway may not majorly impair VSMC function in other vital organs.

In summary, we have uncovered a VSMC-intrinsic, lipid-independent mechanism of plaque stabilization controlled by a KIF13B/KCTD10/KLF4 axis. By promoting KCTD10-mediated KLF4 ubiquitination, KIF13B restrained proinflammatory reprogramming and preserved collagen-rich, cap-stabilizing VSMC states. Pharmacologic inhibition of KLF4 favorably remodeled plaque phenotype in vivo, nominating this pathway as a promising target for reducing residual ASCVD risk by direct stabilization of vulnerable plaques.

## Methods

### Sex as a biological variable.

For animal models, only male mice were used. For clinical samples, both sexes were involved. Sex was not considered as a biological variable.

### Human studies.

Carotid atherosclerotic plaques were obtained from 6 patients undergoing carotid endarterectomy (CEA) at the First Affiliated Hospital of Zhengzhou University between March 2023 and March 2024. Patient enrollment was based on preoperative vascular imaging (CT angiography or carotid duplex ultrasound) confirming significant carotid stenosis, with surgical indication determined by a multidisciplinary vascular team. Written informed consent was obtained from all participants before surgery. This consent approved the use of their tissue specimens and associated clinical data for research, as described in our previous publication (22).

Following resection, each plaque was dissected intraoperatively into anatomically defined segments according to the Athero-Express study protocol (44). For each patient, 2 distinct regions were identified within the same plaque: (a) a culprit segment, typically located at the bifurcation or proximal internal carotid artery, representing the region with the most prominent thickening, calcification, or stenosis as judged by the surgeon and supported by intraoperative assessment; (b) an adjacent upstream segment, located near the cranial base and considered to represent an earlier stage of atherosclerotic involvement. These were designated as the “advanced plaque” and “adjacent control,” respectively. The anatomical classification was conducted independently of histopathological or molecular characteristics and was guided solely by the location and macroscopic appearance at the time of removal.

This segmental sampling strategy enabled paired intraindividual comparisons between different lesion stages within the same patient, thereby reducing interpatient variability. Each pair of advanced and early lesion segments from the same patient was subsequently subjected to parallel downstream analyses.

### Animal studies.

*Kif13b^fl/fl^* mice were generated in our laboratory using the CRISPR/Cas9 gene editing system as described previously (21). Conditional smooth muscle cell–specific *Kif13b*-KO mice (*Kif13b^VSMCKO^*) were generated by pairing *Tagln-Cre* mice (provided by Hongliang Li, Wuhan University, Wuhan, China) with *Kif13b^fl/fl^* mice, and littermates of *Kif13b^fl/fl^* mice were used as controls. *Ldlr*-deficient (*Ldlr^–/–^*) mice were purchased from GemPharmatech.

The *Ldlr^–/–^ Kif13^b–/–^* mouse was generated by crossbreeding of the *Ldlr^–/–^* mouse with the *Kif13b^–/–^* mouse. All experimental animals were bred in an specific pathogen–free–grade (SPF-grade) animal facility with controlled temperature and humidity, a 12-hour light/12-hour dark cycle, and ad libitum access to food and water.

The WD (D12108C) used for the mice in this study was purchased from Research Diets Inc.

Additional details on methods can be found in the Supplemental Methods.

### Statistics.

All statistical analysis was performed using GraphPad Prism version 10.0 (GraphPad Software). All data were presented as the mean ± SEM. Normality of data distribution was assessed using the Shapiro-Wilk test and equality of variance using the Brown-Forsythe test. For comparisons between 2 groups, differences were compared using a 2-tailed, unpaired Student’s *t* test or multiple unpaired *t* tests (normal distribution and equal variance), a Wilcoxon rank-sum test (non-normal distribution), or Welch’s *t* test (unequal variances). For multiple comparisons of equal variance (>2 groups), 1-way ANOVA (single-variable) or 2-way ANOVA (multiple variables) was used, followed by Tukey’s post hoc test (equal variance). The Kruskal-Wallis test and Bonferroni’s post hoc test were used for multiple tests of unequal variance. *P* values of less than 0.05 were considered statistically significant.

### Study approval.

Informed consent was obtained in writing from all participants prior to the collection of human atherosclerotic plaque tissues. The human participant research protocol was reviewed and approved by the Institutional Research Ethics Committee of the First Affiliated Hospital of Zhengzhou University (approval no. 2024-KY-0071). All procedures involving human participants were conducted in accordance with the ethics principles of the Declaration of Helsinki. All animal studies were reviewed and approved by the IACUC of Peking University (protocol no. LA2023460) and were performed in strict adherence to the NIH *Guide for the Care and Use of Laboratory Animals* (8th edition, National Academies Press, 2011).

### Data availability.

The single-cell RNA-seq data were deposited in the NCBI GEO database (GSE314838). Values for all data points are available in the Supporting Data Values file. Additional requests for resources or reagents should be directed to the corresponding author.

## Author contributions

XX and GM were responsible for conceptualization of the project. GM, YH, JC, YZ, YX, ZL, L Zhang, XL, SS, XK, Yahan Liu, and L Zheng designed the study methodology. GZ and JT collected atherosclerotic plaques samples from clinical patients. GM, YH, and JC performed experiments. Yiran Liu, GZ, and SP conducted bioinformatics analysis. GM, YH, and JC performed visualization. XX, ED, and GM acquired funding. XX and ED were responsible for project administration. XX, WH, and YW supervised the study. GM and JC wrote the original draft of the manuscript. XX, JC, and GM wrote, reviewed, and edited the manuscript. All authors read and approved the article.

## Funding support

- National Natural Science Foundation of China (NSFC) grants 82270479, 82070460, and HY2021-1 (to XX).
- Beijing Natural Science Foundation grant 7242084 (to XX).
- Fundamental Research Funds for the Central Universities (to XX).
- Peking University Medicine plus X Pilot Program-Platform Construction Project 2024YXXLHPT010 (to XX).
- CAMS Innovation Fund for Medical Sciences grant 2021-I2M-5-003 (to ED).
- NSFC grant 8240033347 (to GM).

## Supplementary Material

Supplemental data

Unedited blot and gel images

Supporting data values

## Version 1 01/29/2026

In-Press Preview

## Version 2 03/16/2026

Electronic publication

## References

[1] Libby P, Aikawa M (2002). Stabilization of atherosclerotic plaques: new mechanisms and clinical targets. Nat Med.

[2] Falk E (2013). Update on acute coronary syndromes: the pathologists’ view. Eur Heart J.

[3] Kim BK (2022). Long-term efficacy and safety of moderate-intensity statin with ezetimibe combination therapy versus high-intensity statin monotherapy in patients with atherosclerotic cardiovascular disease (RACING): a randomised, open-label, non-inferiority trial. Lancet.

[4] Fiolet ATL (2021). Efficacy and safety of low-dose colchicine in patients with coronary disease: a systematic review and meta-analysis of randomized trials. Eur Heart J.

[5] Nidorf SM (2024). Low-dose colchicine for atherosclerosis: long-term safety. Eur Heart J.

[6] Patrono C (2024). Low-dose aspirin for the prevention of atherosclerotic cardiovascular disease. Eur Heart J.

[7] Gomez-Delgado F (2024). Residual cardiovascular risk: when should we treat it?. Eur J Intern Med.

[8] Basatemur GL (2019). Vascular smooth muscle cells in atherosclerosis. Nat Rev Cardiol.

[9] Pan H (2020). Single-cell genomics reveals a novel cell state during smooth muscle cell phenotypic switching and potential therapeutic targets for atherosclerosis in mouse and human. Circulation.

[10] Zhang F (2021). An update on the phenotypic switching of vascular smooth muscle cells in the pathogenesis of atherosclerosis. Cell Mol Life Sci.

[11] Miano JM (2021). Fate and state of vascular smooth muscle cells in atherosclerosis. Circulation.

[12] Miao G (2022). Vascular smooth muscle cell c-Fos is critical for foam cell formation and atherosclerosis. Metabolism.

[13] Alencar GF (2020). Stem cell pluripotency genes Klf4 and Oct4 regulate complex SMC phenotypic changes critical in late-stage atherosclerotic lesion pathogenesis. Circulation.

[14] Yap C (2021). Six shades of vascular smooth muscle cells illuminated by KLF4 (Krüppel-like factor 4). Arterioscler Thromb Vasc Biol.

[15] Cho DI (2023). ANGPTL4 stabilizes atherosclerotic plaques and modulates the phenotypic transition of vascular smooth muscle cells through KLF4 downregulation. Exp Mol Med.

[16] Shankman LS (2015). KLF4-dependent phenotypic modulation of smooth muscle cells has a key role in atherosclerotic plaque pathogenesis. Nat Med.

[18] Kanai Y (2014). KIF13B enhances the endocytosis of LRP1 by recruiting LRP1 to caveolae. J Cell Biol.

[19] Waters SB (2021). VEGFR2 trafficking by KIF13B is a novel therapeutic target for wet age-related macular degeneration. Invest Ophthalmol Vis Sci.

[20] Chen J (2025). Macrophage-derived KIF13B interacts with USP9X to attenuate abdominal aortic aneurysm development by potentiating TFEB stability. Theranostics.

[21] Miao GL (2025). Motor protein KIF13B orchestrates hepatic metabolism to prevent metabolic dysfunction-associated fatty liver disease. Mil Med Res.

[22] Xu Y (2025). The macrophage-derived motor protein KIF13B enhances MERTK-mediated efferocytosis and prevents atherosclerosis in mice. Eur Heart J.

[23] Tabaei S, Tabaee SS (2019). DNA methylation abnormalities in atherosclerosis. Artif Cells Nanomed Biotechnol.

[24] Zhang L (2023). DNA methylation and histone post-translational modifications in atherosclerosis and a novel perspective for epigenetic therapy. Cell Commun Signal.

[25] Taylor JCK (2025). Delineation of a thrombin receptor-stimulated vascular smooth muscle cell transition generating cells in the plaque-stabilizing fibrous cap. Cardiovasc Res.

[26] Lonardo A (2018). Hypertension, diabetes, atherosclerosis and NASH: Cause or consequence?. J Hepatol.

[27] Zhang H (2025). Hypercholesterolemia-induced LXR signaling in smooth muscle cells contributes to vascular lesion remodeling and visceral function. Proc Natl Acad Sci U S A.

[28] Halmos B (2025). SMC Abca1 and Abcg1 deficiency enhances urinary bladder distension but not atherosclerosis. Circ Res.

[29] Houweling AC (2019). Loss-of-function variants in myocardin cause congenital megabladder in humans and mice. J Clin Invest.

[30] Hu X (2025). Elucidating the role of KCTD10 in coronary atherosclerosis: Harnessing bioinformatics and machine learning to advance understanding. Sci Rep.

[31] Chen R (2023). Phenotypic switching of vascular smooth muscle cells in atherosclerosis. J Am Heart Assoc.

[32] Wirka RC (2019). Atheroprotective roles of smooth muscle cell phenotypic modulation and the TCF21 disease gene as revealed by single-cell analysis. Nat Med.

[33] Mosquera JV (2023). Integrative single-cell meta-analysis reveals disease-relevant vascular cell states and markers in human atherosclerosis. Cell Rep.

[34] Shen Y (2022). BMAL1 modulates smooth muscle cells phenotypic switch towards fibroblast-like cells and stabilizes atherosclerotic plaques by upregulating YAP1. Biochim Biophys Acta Mol Basis Dis.

[35] Pan H (2024). Atherosclerosis is a smooth muscle cell-driven tumor-like disease. Circulation.

[36] Jaiswal S (2017). Clonal hematopoiesis and risk of atherosclerotic cardiovascular disease. N Engl J Med.

[37] Gumuser ED (2023). Clonal hematopoiesis of indeterminate potential predicts adverse outcomes in patients with atherosclerotic cardiovascular disease. J Am Coll Cardiol.

[38] Jaiswal S, Libby P (2020). Clonal haematopoiesis: connecting ageing and inflammation in cardiovascular disease. Nat Rev Cardiol.

[39] Zhou Q (2024). The CRL3^KCTD10^ ubiquitin ligase-USP18 axis coordinately regulates cystine uptake and ferroptosis by modulating SLC7A11. Proc Natl Acad Sci U S A.

[40] Yin Z (2025). KCTD10 inhibits lung cancer metastasis and angiogenesis via ubiquitin-mediated β-catenin degradation. Front Immunol.

[41] Arsenault BJ (2011). Lipid parameters for measuring risk of cardiovascular disease. Nat Rev Cardiol.

[42] Wong ND (2017). Residual atherosclerotic cardiovascular disease risk in statin-treated adults: the multi-ethnic study of atherosclerosis. J Clin Lipidol.

[43] Yeo M (2021). Repurposing cancer drugs identifies kenpaullone which ameliorates pathologic pain in preclinical models via normalization of inhibitory neurotransmission. Nat Commun.

[44] Verhoeven BA (2004). Athero-express: differential atherosclerotic plaque expression of mRNA and protein in relation to cardiovascular events and patient characteristics. Rationale and design. Eur J Epidemiol.

