# Supplementary material for: Vascular smooth muscle cell–derived KIF13B inhibits proinflammatory responses to protect against atherosclerosis
Source: J Clin Invest. 2026 Jan 29;136(6):e194175. doi: 10.1172/JCI194175 (PMC12987658; doi:10.1172/JCI194175)
Supplement: Supplemental data [file jci-136-194175-s268.pdf]

Supplemental Methods

Atherosclerosis-prone Animal Models

Kif13b$^{flox/flox}$ mice and *Kif13b*^{VSMCKO} mice intravenously administered AAV8-PCSK9-D377Y (1E11 vg/mouse), *Ldlr*$^{-/-}$ and *Ldlr*$^{-/-}$*Kif13b*$^{-/-}$ mice were used in the study.

To establish a mouse model of atherosclerosis, 8- to 10-week-old male mice were selected and given a WD for 20 weeks. The mice were then anaesthetized, and the aorta and cardiac outflow tracts were harvested.

Kenpaullone treatment in mice also used *Kif13b*$^{flox/flox}$ mice and *Kif13b*^{VSMCKO} mice intravenously administered AAV8-PCSK9-D377Y (1E11 vg/mouse), which were first fed an 8-week WD, followed by gavage with 1 mg/kg/day of Kenpaullone (HY-12302, MCE) or vehicle and then continued with the WD for an additional 12 weeks. The mice were then anaesthetized, and the aorta and cardiac outflow tracts were harvested.

Blood Collection and Biochemical Analysis

Blood samples were collected from the retro-orbital plexus of mice after a 4-hour fasting period, using anticoagulant-treated tubes as required by the experimental design. Plasma was isolated by centrifugation at 4 °C for 10 min and stored at -80 °C until further use.

Plasma total cholesterol (TC; catalog no. 100060090) and triglyceride (TG; catalog no. 100060100) levels were determined using commercial assay kits from BIOSINO BIO-TECHNOLOGY & SCIENCE INC (Beijing, China). Alanine aminotransferase (ALT; catalog no. C009-2-1) and aspartate aminotransferase (AST; catalog no. C010-2-1) activities were measured with corresponding kits supplied by NJJCBIO (Nanjing, China), in accordance with the manufacturers' protocols. Plasma cytokine concentrations were assessed using enzyme-linked immunosorbent assay (ELISA) kits from RUIXIN BIOTECH (Quanzhou, China), following the manufacturer’s instructions: Mouse IL-1β (RXW203063), Mouse IL-6 (RXW203049), and Mouse TNF-α (RX201248M).

Characterization of Atherosclerotic Lesions

Fresh-frozen tissues embedded in OCT compound were sectioned at a thickness of 7 μm, starting from the level of the aortic valves and extending through the ascending aorta. Sections were systematically collected onto eight slides. The collection began when the first two aortic valve leaflets became visible and continued until the aortic wall was no longer observable.

For each experimental animal, one slide with eight consecutive sections was stained with hematoxylin (Sigma, HHS128-4L) and eosin (Sigma, HT110232-1L) for morphological evaluation. To ensure standardized lesion quantification across different animals, the section at which all three aortic valve leaflets disappeared was designated as the transition point (“0”). This point marked the end of the aortic sinus and the start of the ascending aorta.

Lesion areas were evaluated on three sections before, at, and three sections after this reference point. As a result, a total of eight sections per aortic root were analyzed. The mean lesion area across these sections was utilized for intergroup comparisons. Atherosclerotic lesions in the aortic root were quantitatively analyzed on serial sections. The quantification was performed using Nikon NIS-Elements software (version 5.11.00), with the lesion boundaries manually delineated in accordance with the guidelines of the American Heart Association (AHA).

Plaque composition and vulnerability were evaluated through multiple staining techniques. Hematoxylin and eosin (H&E) staining was employed to examine general morphology. Oil Red O (ORO) staining was utilized to detect lipid deposition. Anti-CD68 antibody staining was carried out to assess macrophage infiltration, while anti-α-smooth muscle actin (α-SMA) antibody staining was used to analyze VSMCs. Terminal deoxynucleotidyl transferase dUTP nick end labeling (TUNEL) staining was performed to identify apoptotic cells, and Masson's trichrome staining was applied to evaluate collagen fibrosis. The quantitative results were presented as the percentage of the positively stained area within the plaques. For the en face analysis of the entire

aorta stained with ORO, atherosclerotic lesions were quantified along the medial aspect.

Cell Culture and Treatment

Primary human aortic smooth muscle cells (HASMCs) were obtained from Procell (CP-H081, China) and cultured in complete medium (CM-H081, Procell, China) according to the manufacturer's instructions. For all experiments, cells at passages 2-5 were used.

To induce phenotypic modulation, HASMCs were subjected to serum starvation for 12 hours. Subsequently, they were treated with or without 50 μg/mL oxidized low-density lipoprotein (ox-LDL) for 24 hours before RNA extraction and 48 hours before protein extraction. To assess KLF4 protein stability, HASMCs were treated with the protein synthesis inhibitor cycloheximide (CHX; 50 μg/mL; HY-12320, MCE) or the proteasome inhibitor MG132 (10 μM; HY-13259, MCE). To suppress KLF4 protein expression, HASMCs were treated with Kenpaullone (10 μM).

Preparation and Application of Lentivirus

Lentiviral vectors (15 mg), along with pMDLg/pRRE, RSV/Rev, and VSV-G (5 mg each), were co-transfected into 293T cells using the calcium phosphate ($Ca_3(PO_4)_2$) method in 10-cm dishes. The cells were incubated for 12-16 hours, after which the medium was replaced. Two days post-transfection, the viral supernatant was collected and filtered through 0.45 μm filters (SLHP033RB, Millipore). The filtered supernatant was concentrated to 1x10^8 TU through ultracentrifugation and then stored at -80°C, for in vivo and in vitro experiments in the future.

RNA Interference

The siRNA sequences targeting KIF13B (si*KIF13B*) and KLF4 (si*KLF4*), along with a scrambled control, were commercially synthesized by Sangon Biotech (Shanghai, China). The corresponding oligonucleotide sequences are provided in Supplementary Table 1 and 2.

For transfection, HASMCs were seeded into 6-well plates in complete medium

and transfected with siRNA using Lipofectamine RNAiMAX reagent (13778150, Invitrogen), following the manufacturer's guidelines. To assess knockdown efficiency, cells were collected 48 hours post-transfection for subsequent analysis.

Plasmid Transfection

HA-tagged expression constructs, including full-length *KIF13B* plasmid, *KIF13B* motor domain truncated plasmid, and *KCTD10* plasmid were synthesized by Sangon Biotech (Shanghai, China). Transfection was performed in HASMCs using Lipofectamine 3000 (L3000015, Invitrogen, American) according to the manufacturer's protocol. Cells were harvested 48 hours after transfection for subsequent analyses.

Immunofluorescent Staining

Frozen tissue sections (7 μm thick) were fixed in 4% PFA for 15 minutes at RT, followed by permeabilization with 0.3% Triton X-100 for 10 minutes at RT, and then embedded in PBS containing 10% donkey serum and incubated with anti-KIF13B (an in-house prepared antibody, antigen sequence: CQTLKVREHSVLGPYVDGLSK), anti-α-SMA (BM0002, Boster), anti-Cluster of Differentiation 68 (CD68) (BA3638, Boster), and anti-KCTD10 (27279-1-AP, Proteintech) primary antibodies (1:200) were incubated overnight at 4°C. Normal isotype IgG (Rabbit, 1:200, 3900, Cell Signaling Technology; Mouse, 1:200, 5415, Cell Signaling Technology) was used as a negative control. After a rinse with PBS 3 times, Alexa Fluor 555 (1:1000, ab150062, Abcam), 488 (1:1000, ab150113, Abcam), or 647 (1:1000, ab150107 or ab150083, Abcam) -conjugated secondary antibodies were incubated for 1 h at 37°C in the dark. Sections were made using an anti-fade mounting medium containing DAPI (ZLI9557, ZSGB-BIO) to stain cell nuclei.

Cells seeded on glass coverslips were fixed with 4% paraformaldehyde for 10 min, and permeabilized with 0.2% (v/v) Triton X-100 in PBS for 10 min. After blocking with 10% donkey serum in PBS, samples were incubated with anti-KLF4 antibody (1:200, 11880-1-AP, Proteintech) overnight at 4 °C, using normal isotype IgG (1:200,

#5415, Cell Signaling Technology) as a negative control. Following three washes with PBS, Alexa Fluor 647-conjugated secondary antibodies (1:1000, ab150107, Abcam) were applied and incubated at 37 °C for 1 h in the dark. Cell nuclei were counterstained with DAPI (C1005, Beyotime).

Super-resolution fluorescence imaging was performed by an integrated fluorescence microscope BZ-X810 (KEYENCE, America) or a confocal microscope FV3000 (Olympus, Japan). Immunofluorescence staining was quantified using Image J 10.0.

Vascular function studies

The thoracic aorta from *Kif13b^{VSMCKO} and Kif13b$^{flox/flox}$* mice was isolated and placed in cold Krebs bicarbonate solution (120.4 mM NaCl, 5.9 mMKCl, 2.5 mM CaCl2, 1.2 mM MgCl2, 1.2 mM NaH2PO4, 11.5 mM glucose, 25 mM NaHCO3), cleaned of adhering tissue and cut into equally sized aortic rings (2 mm in length) at the exact same location of each aorta. Aortic rings were mounted on 200 μm stainless steel pins in individual organ baths of a Mulvany myograph (Model 610M, Danish Myo Technology). Briefly, organ baths containing 6 mL of Krebs solution were maintained at 37°C and continuously aerated with 95% O_2 - 5% CO_2 to maintain a pH at 7.4. Aortic rings were equilibrated for 40 min until they were at a steady state baseline in terms of isometric force and then stretched to a transmural pressure of 13.3 kPa using the proprietary DMT Normalization Procedure software module. Rings were primed and checked for viability by two consecutive stimulations with a high concentration of potassium chloride (KCl, 60 mM), followed by intermediate washes using Krebs buffer, and renewed stabilization. To study vasoconstriction, aortic rings were stimulated with cumulative concentrations of phenylephrine (1 nM - 100 μM) (P6126; Sigma-Aldrich). Contractions induced by phenylephrine or KCl were corrected by the vessel size (in mm) and expressed as mN/mm. Nonlinear regression was used to analyze the concentration-response curves for phenylephrine.

Oral fat load test

Mice were starved for 4 h and weighed before blood was collected during fasting. Then these mice were gavaged with olive oil (10 ul/g, 8011845004871, La Sicilia), and blood was collected from the intra-orbital vein at 30 min, 1 h, 2 h, and 4 h after the olive oil challenge.

Magnetic resonance imaging (MRI)

The bladder volume was measured in mice fed with WD for 8 weeks by magnetic resonance imaging (MRI). MRI was performed using a 3.0 T MAGNETOM Trio (Siemens, Germany), with a mouse body coil (CG, China). Mice were anesthetized with 1.5% isoflurane (R510-22, RWD, China) in 1 L/min of compressed air. The MRI protocol contains two sequences. Firstly, a T2-weighted rapid acquisition relaxation enhanced sequence was acquired to obtain a coronal image (TR = 2050 ms, TE = 63.1 ms, NEX = 1, slice thickness = 2 mm, slices = 19, FOV = 13 × 13 cm^2, matrix = 384 × 384, acquisition time = 1:30 min). Secondly, a t2-weighted rapid acquisition relaxation enhanced sequence (TR =2050 ms, TE = 70.8 ms, NEX = 1, slice thickness = 2 mm, slices = 19, FOV = 13 × 13 cm^2, matrix = 384 × 384, acquisition time = 1:30 min) was acquired to obtain a cross-sectional image. Measurement of bladder tibia with the Bee Viewer APP.

RNA Isolation and Quantitative PCR (qPCR)

Total RNA was isolated from tissues or cultured cells using Trizol reagent (ET111-01-V2, TransGen, China). Subsequently, 5 μg of RNA was reverse-transcribed into cDNA with a commercial synthesis kit (AT321-01, TransGen, China). Quantitative PCR was performed using Top Green qPCR SuperMix (AQ132-24, TransGen, China) on a QuantStudio 3 real-time PCR system (Thermo Fisher Scientific). Glyceraldehyde-3-phosphate dehydrogenase (GAPDH) was used as the endogenous control for normalization. Primer sequences are listed in Supplementary Table 3 and 4.

Protein Extraction and Western Blot

Protein extracts were prepared from abdominal aortic tissues or cultured cells

using ice-cold RIPA lysis buffer (DE301-1, Di-Ning) supplemented with protease inhibitor cocktail (04693116001, Roche). Protein concentration was determined using a BCA assay kit (23225, Thermo Fisher Scientific). Lysates were mixed with 5× SDS loading buffer (P0015L, Beyotime), denatured at 95 °C for 10 min, separated by SDS-PAGE, and transferred onto nitrocellulose membranes. Membranes were blocked with 5% BSA in TBST (25 mM Tris-HCl, 137 mM NaCl, 2.7 mM KCl, 0.075% Tween-20, pH 7.4) for 1 hour at room temperature, followed by incubation at 4 °C overnight with the following primary antibodies: anti-KIF13B (SAB2101257, Sigma), anti-TAGLN (10493-1-AP, Proteintech), anti-fibroblast-specific protein 1 (FSP1) (YP-mAb-04501, UpingBio), anti-KLF4(YP-Ab-02931, UpingBio), anti-KLF4 antibody (11880-1-AP, Proteintech) anti-KCTD10 (YP-mAb-11879, UpingBio), anti-UB (58395, Cell Signaling Technology), anti-GAPDH (60004-1-Ig, Proteintech) primary antibodies were incubated overnight at 4°C. After washing three times with TBST, membranes were incubated for 1 h at room temperature with horseradish peroxidase-conjugated secondary antibody (ZSGB-BIO, China) diluted in TBST containing 5% BSA. Protein bands were visualized using an enhanced chemiluminescence substrate (36208ES, Yeasen) on an iBright Imaging System (CL1500, Invitrogen, USA).

Coimmunoprecipitation (Co-IP) Assay

Cells were lysed on ice using RIPA buffer supplemented with protease inhibitors. Lysates were centrifuged at high speed to collect the supernatant, and protein concentration was determined. Equal amounts of protein were incubated overnight at 4 °C with anti-KLF4 (11880-1-AP, Proteintech, USA), anti-KCTD10(YP-mAb-11879, UpingBio, China), or control IgG antibody (AC005, ABclonal, China) together with protein A/G agarose beads (R8281, Solarbio, China). The immunoprecipitated proteins were washed three times with RIPA buffer, and bound proteins were eluted by boiling in 2× SDS-PAGE loading buffer at 95 °C for 5 min. The immunocomplexes were then separated by SDS-PAGE.

Enzyme-linked immunosorbent assay

The supernatant of HASMCs culture was collected to detect cytokines concentration using ELISA kits from RUIXIN BIOTECH (Quanzhou, China) following the manufacturer's instructions: Human IL-1β ELISA kit (RXM106152H), Human IL-6 ELISA kit (RXM106126H), Human CCL2 ELISA kit (RXM104010H), Human Col1α1 ELISA kit (RX2D2030846), Human Col2α1 ELISA kit (RXM2D166656), Human Col3α1 ELISA kit (RXM100781H).

Collagen gel contraction assay

The collagen gel contraction assay was performed using a commercial Cell Contraction Assay Kit (CBA-021, Cell Biolabs, San Diego, CA, USA), following the manufacturer's protocol. In brief, HASMCs transfected with either *Scramble* (20 nM) or *siKIF13B* for 48 hours were resuspended in DMEM supplemented with 10% fetal bovine serum (FBS) at a density of 5×10^5 cells/mL. A collagen lattice was prepared by combining the cell suspension with ice-cold collagen gel solution at a 1:4 volume ratio. Then, 0.5 mL of the mixture was added to each well of a 24-well plate and allowed to polymerize at 37 °C for 1 hour. Subsequently, 1.0 mL of DMEM containing 10% FBS was gently overlaid onto each gel. After 24 hours of incubation, the plates were scanned, and gel area was quantified using Fiji software.

Transfection and dual luciferase assays

Transcriptional activity of KLF4 was assessed using a dual-luciferase reporter assay following treatment with Kenpaullone (n = 9 per group). HASMCs were seeded into 12-well plates and co-transfected with 500 ng of pGL3-KLF4 promoter construct and 50 ng of pTK-Renilla plasmid for 24 hours. Cells were then incubated with 10 μM Kenpaullone for 4 hours, after which luciferase activity was measured using a dual-luciferase assay kit (11402ES60, Yeasen, China). Firefly luciferase activity was normalized to Renilla luciferase activity and expressed as relative luciferase units (RLU).

RNA-Seq data analysis

The RNA-Seq data were first used for quality check and filtered by trim_galore (https://github.com/FelixKrueger/TrimGalore) and then mapped to the GRCm38 reference sequence by Hisat2. We then used HTSeq to calculate the expression matrix(1). Vst normalization was performed by the DESeq2 package(2). P-value was calculated by one-tail Wilcoxon rank sum test based on normalized KIF13B expression value.

Single-cell RNA-Seq analysis

The Python package scanpy was used to perform upstream single-cell RNA-Seq analysis(3). After filtering cells and genes, we used the scVI package to remove batch effect and perform data embedding, followed by a manual selection of cell type markers to annotate cell types(4). The cell proportion analysis was conducted by the pertpy (https://github.com/scverse/pertpy) package. The VSMC-Fibs were isolated for further downstream analysis. The GSEA analysis was performed by the GSEApy package(5). monocle2 was used for pseudo-time inference and cell trajectory construction. Branched expression analysis modeling was used to find DEGs in different branches(6). The Python package pySCENIC was used to infer Gene Regulatory Networks (GRNs) in VSMC-Fib(7). Regulon active score was calculated by the AUCell function and further binarized for comparison.

RNA microarray data analysis

The public microarray data were collected from the GEO database (GSE120521) by the GEOquery (http://seandavi.github.io/GEOquery/) R package. We first performed PCA analysis to detect and remove anomalous samples to get accurate DEGs. The R package limma was used to normalize data and get DEGs between stable and unstable atherosclerotic plaque tissues(8).

DNA methylation microarray data analysis

The public methylation microarray data were collected from the GEO database (GSE149759, GSE46401, and GSE66500). Beta value matrix was concatenated for GSE149759 and GSE46401 for further analysis. Limma package was used for batch

effect remove and differentially expressed probes calculation. Infinium Human Methylation 450K BeadChip reference was used to align different probes. The middle value of the same probe was calculated for comparison between different groups. Gviz package was used to visualize the profile plot in the *KIF13B* gene body(9). P-value was calculated by one-tail Wilcoxon Rank Sum test.

Protein Omics data analysis

The Protein Omics data were collected from the ProteomeXchange dataset (PXD062283). Promor package was used for downstream data imputation and normalization(10). The minDet algorithm was used for data imputation and the limma package was used for differentially expressed protein calculation. P-value was calculated by the Likelihood Ratio test.

Spatial transcriptome data analysis

The spatial transcriptome data were collected from the GEO database (GSE274572). The upstream analysis was the same as single-cell RNA-seq analysis. After clustering and cell type annotation, the spatial plot was colored by cell type.

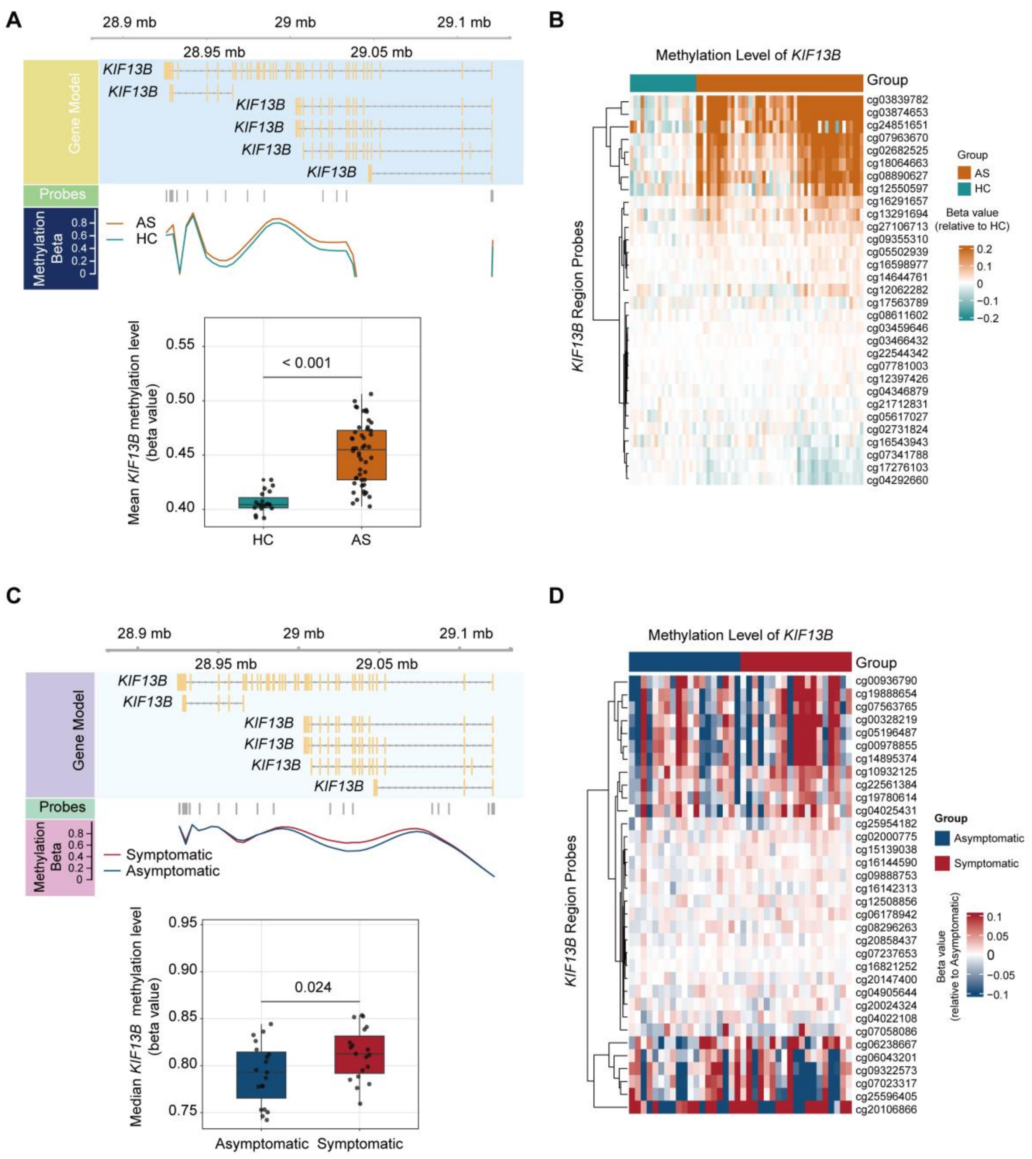

Supplemental Figure 1. DNA methylation of KIF13B is elevated in human atherosclerotic plaques

(**A**) Methylation profile and quantification of *KIF13B* methylation levels in atherosclerotic (n = 48) versus healthy arterial tissues (n = 19) from GEO database (GSE46394 and GSE149759). (**B**) Heatmap of *KIF13B* methylation levels in different sites between atherosclerotic (n = 48) and healthy arterial tissues (n = 19) from GEO database (GSE46394 and GSE149759). (**C**) Methylation profile and quantification of *KIF13B* methylation levels in symptomatic versus asymptomatic atherosclerotic plaque tissues (n = 19 per group) from GEO database (GSE66500). (**D**) Heatmap of *KIF13B* methylation levels in different sites between symptomatic versus

asymptomatic atherosclerotic plaque tissues (n = 19 per group) from GEO database (GSE66500).

Data are presented as the mean ± SEM and were analyzed by two-tailed Wilcoxon rank-sum test (**A**, **C**).

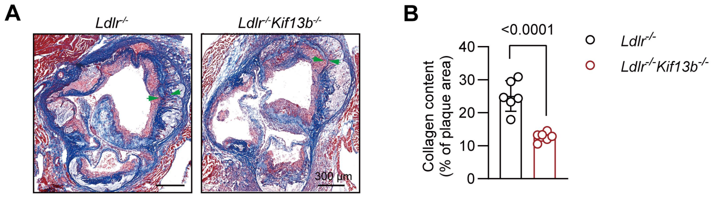

Supplemental Figure 2. Loss of *Kif13b* accelerates atherosclerotic plaque formation with reduced collagen content in Western diet-fed *Ldlr*-deficient mice

Eight-week-old male *Ldlr*$^{-/-}$ and *Ldlr*$^{-/-}$*Kif13b*$^{-/-}$ mice were fed a Western diet (WD) for 20 weeks and subsequently euthanized. Heart tissues were harvested, and the aortic root were processed for frozen-sectioning and then Masson's staining. Blue staining indicates collagen, and the green arrowheads show the thickness of the fibrous cap in the atherosclerotic plaque. (**A**) Representative Masson's trichrome-stained images of the aortic root. (**B**) Quantitative analyses of the percentage of collagen content in plaque area (n = 6 per group).

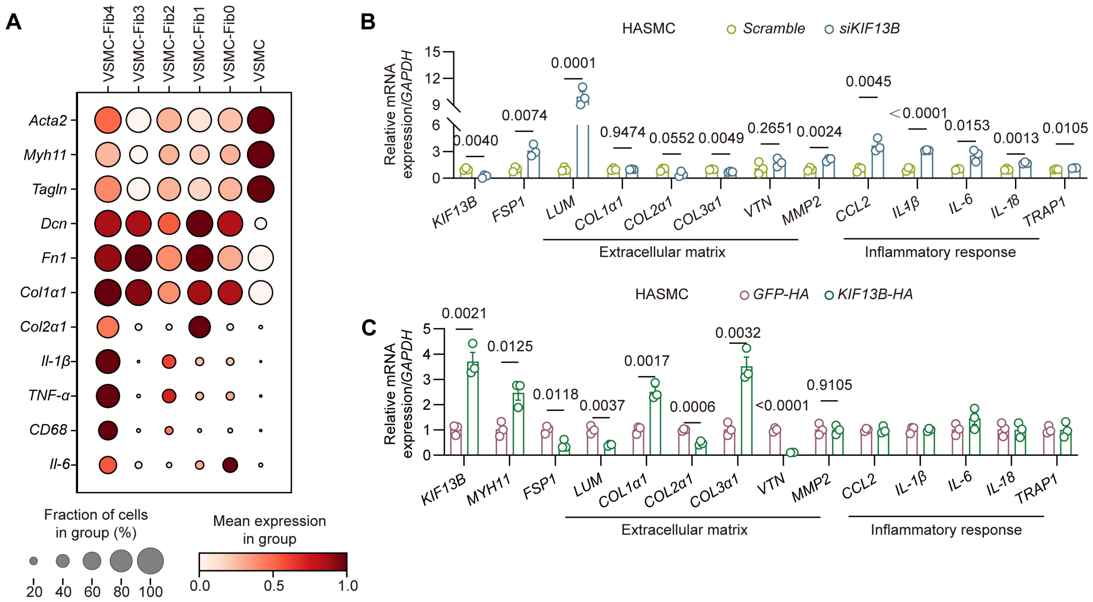

Supplemental Figure 3. KIF13B regulates VSMCs phenotype switch

(**A**) Dot plot showing the expression of cell type markers in VSMC-Fibs clusters, dots coloured by min-max scaled gene expression level. (**B**) QPCR analysis of the expression of genes related to VSMC-Fibs phenotypic switch (*FSP1*, *LUM*), collagen production (*COL1α1*, *COL2α1*, *COL3α1*), and inflammatory factor (*CCL2*, *IL-1β*, *IL-6*) in HASMCs transfected with*Scramble* or *siKIF13B* for 24 hours (n = 3 independent biological replicates). (**C**) QPCR analysis the expression of genes related to VSMC-Fibs phenotypic switch (*FSP1*, *LUM*), collagen production (*COL1α1*, *COL2α1*, *COL3α1*), and inflammatory factor (*CCL2*, *IL-1β*, *IL-6*) in HASMCs infected with lentivirus (LV) expressing *GFP-HA* or *KIF13B-HA* for 24 hours (n = 3 independent biological replicates).

Data are presented as the mean ± SEM and were analyzed by multiple unpaired t tests (**B**, **C**).

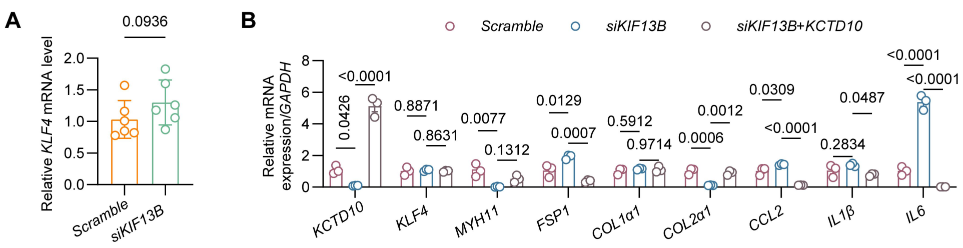

Supplemental Figure 4. Inactivation of KIF13B enhances pro-inflammatory VSMC reprogramming through blocking KCTD10-mediated KLF4 ubiquitination

(**A**) *KLF4* mRNA levels in HASMCs transfected with *Scramble* or *siKIF13B* and subsequently treated with oxLDL (50 μg/ml) for 24 hours (n = 6 independent biological replicates). (**B**) QPCR analysis of the expression of genes related to VSMC-Fibs phenotypic switch (*FSP1*, *MYH11*), collagen production (*COL1α1*, *COL2α1*), and inflammatory factor (*CCL2*, *IL-1β*, *IL-6*) in HASMCs transfected with *Scramble* or *siKIF13B* in the presence or the absence of *KCTD10* overexpression and subsequently treated with oxLDL (50 μg/ml) for 24 hours (n = 3 independent biological replicates).

Data are presented as the mean ± SEM and were analyzed by two-tailed unpaired Student's t-test (**A**) or one-way ANOVA with Tukey post hoc tests (**B**).

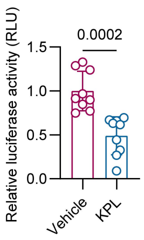

Supplemental Figure 5. Kenpaullone inhibites the promoter activity of KLF4 in HASMCs

Transcriptional activity of KLF4 was assessed using a dual-luciferase reporter assay following a treatment with or without Kenpaullone (n = 9 independent biological replicates). HASMCs were seeded into 12-well plates and co-transfected with 500 ng of pGL3-KLF4 promoter construct and 50 ng of pTK-Renilla plasmid for 24 hours. Cells were then incubated with 10 μM Kenpaullone for 4 hours, followed by the measurement of luciferase activity using a dual-luciferase assay kit. Firefly luciferase activity was normalized to Renilla luciferase activity and expressed as relative luciferase units (RLU). KPL: Kenpaullone.

Data are presented as the mean ± SEM and were analyzed by two-tailed unpaired Student's t-test.

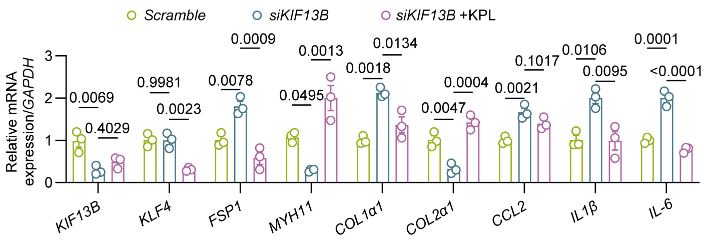

Supplemental Figure 6. Kenpaullone reverses the phenotypic switching of HASMCs induced by KIF13B deficiency

QPCR analysis of the expression of genes related to VSMC-Fibs phenotypic switch (*FSP1*, *MYH11*), collagen production (*COL1α1*, *COL2α1*), and inflammatory factor (*CCL2*, *IL-1β*, *IL-6*) in HASMCs transfected with *Scramble* or *siKIF13B* with or without Kenpaullone treatment (10 μM) and subsequently treated with oxLDL (50 μg/ml) for 24 hours (n = 3 independent biological replicates). KPL: Kenpaullone.

Data are presented as the mean ± SEM and were analyzed by one-way ANOVA with Tukey post hoc tests.

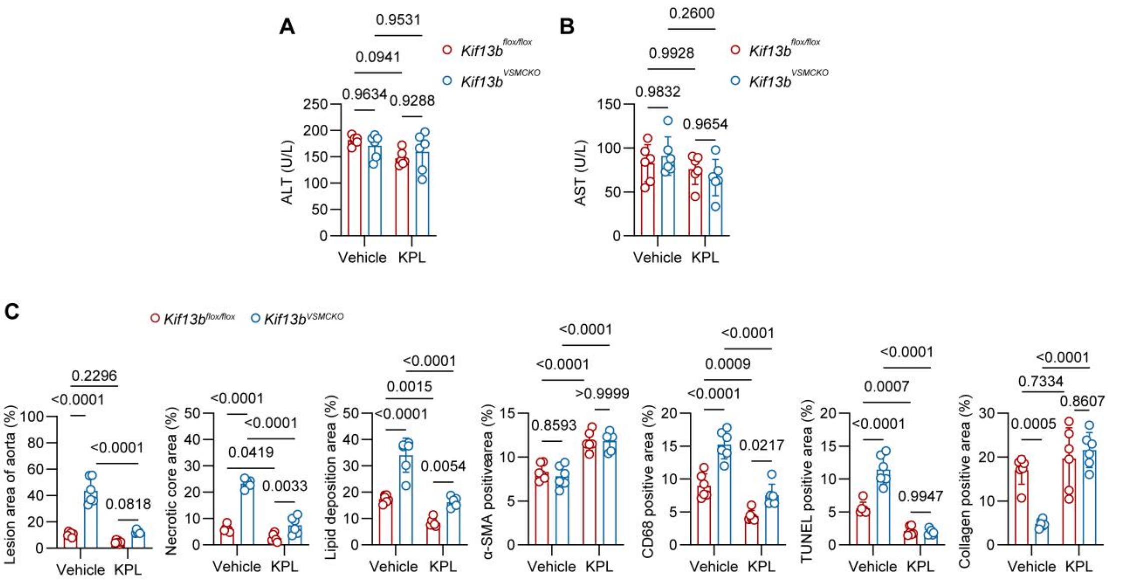

Supplemental Figure 7. Kenpaullone ameliorates atherosclerosis without inducing liver toxicity

8-week-old male *Kif13b*$^{flox/flox}$ and *Kif13b*^{VSMCKO} mice were injected with AAV8-PCSK9-D377Y via the tail vein for 2 weeks and then fed a WD for 20 weeks to establish advanced atherosclerosis, followed by an oral administration of Kenpaullone (1 mg/kg/d) for the last 12 weeks (n = 6 per group).

(**A** and **B**) Plasma alanine transaminase (ALT) (**A**) and aspartate transferase (AST) (**B**) were detected in the indicated mice. (**C**) Quantitative analyses: the percentage of lesion areas in the entire aorta, the relative areas of the necrotic core, CD68-positive staining, αSMA-positive staining and TUNEL-positive staining within the aortic root sections as well as lipid deposition areas of aortic roots, and the percentage of collagen contents in plaque areas.

Data are presented as the mean ± SEM and were analyzed by two-way ANOVA with Tukey post hoc tests (**A**-**C**).

Supplemental Table 1. *siRNA* sequences for Human *KIF13B* gene

Gene name	Sequences	
	Sense (5’ -3’)	Anti-sense (5’ -3’)
si*KIF13B*-1	CCUCCAUGAAGAACGAGAAUATT	UAUUCUCGUUCUUCAUGGAGGTT
si*KIF13B*-2	CCCAGUAAUACGAUCAUACUUTT	AAGUAUGAUCGUAUUACUGGGTT
si*KIF13B*-3	GCCUUGAAGAUCUGCGACAAATT	UUUGUCGCAGAUCUUCAAGGCTT

Supplemental Table 2. *siRNA* sequences for Human *KLF4* gene

Gene name	Sequences	
	Sense (5' -3')	Anti-sense (5' -3')
si*KLF4*-1	GAGAGACCGAGGAG	UUGAACUCCUCGGU
si*KLF4*-2	GGAGAAGACACUGC	UUGACGCAGUGUCU
si*KLF4*-3	GGACUUUAUUCUCU	AUUGGAGAGAAUAA

Supplemental Table 3. The list of human primer sequences

Gene name	Forward primer	Reverse primer
IL1B	GGACAGGATATGGAGCAACAAG	TTCAACACGCAGGACAGGTA
IL6	CTTCGGTCCAGTTGCCTTCT	GGCTTGTTCCTCACTACTCTCA
CCL2	AAAGTCTCTGCCGCCCTTC	CTTGCTGCTGGTGATTCTTCTA
KLF4	CCACACTGCCAGAAGAGAATT	GACTCACCAAGCACCATCATT
FSP1	TGTCCACCTTCCACAAGTA	TGTCCAAGTTGCTCATCAG
TAGLN	CGGCAGCATCATACCAATCG	GGCATCCTCTGACTCCACTC
MYH11	GGCAACTCGTGTCCAACCT	GCGTCTTCATCTCCTCCATCT
COL1α1	TCGGAGGAGAGTCAGGAAGG	CAGCAACACAGTTACACAAGGA
COL2α1	ATGGAGACTGGCGAGACTTG	GCAGGCGTAGGAAGGTCAT
LUM	CATCCATCTCCAGCACAATCG	TCCACTATCAGCCAGTTCGTT
GAPDH	GGAGCGAGATCCCTCCAAAAT	GGCTGTTGTCATACTTCTCATGG

Supplemental Table 4. The list of mouse primer sequences

Gene	Forward primer	Reverse primer
Kif13b	AGGAGGGCAGCAACATCAACAA	TGTCCTTGAGCAGCCATGTGAG
Gapdh	CCAAGGTCATCCATGACAACTT	AGGGGCCATCCACAGTCTT

References

1. Anders S, Pyl PT, and Huber W. HTSeq--a Python framework to work with high-throughput sequencing data. *Bioinformatics.* 2015;31(2):166-9.
2. Love MI, Huber W, and Anders S. Moderated estimation of fold change and dispersion for RNA-seq data with DESeq2. *Genome Biol.* 2014;15(12):550.
3. Wolf FA, Angerer P, and Theis FJ. SCANPY: large-scale single-cell gene expression data analysis. *Genome Biol.* 2018;19(1):15.
4. Lopez R, Regier J, Cole MB, Jordan MI, and Yosef N. Deep generative modeling for single-cell transcriptomics. *Nat Methods.* 2018;15(12):1053-8.
5. Fang Z, Liu X, and Peltz G. GSEApy: a comprehensive package for performing gene set enrichment analysis in Python. *Bioinformatics.* 2023;39(1).
6. Ozadam H, Tonn T, Han CM, Segura A, Hoskins I, Rao S, et al. Single-cell quantification of ribosome occupancy in early mouse development. *Nature.* 2023;618(7967):1057-64.
7. Van de Sande B, Flerin C, Davie K, De Waegeneer M, Hulselmans G, Aibar S, et al. A scalable SCENIC workflow for single-cell gene regulatory network analysis. *Nat Protoc.* 2020;15(7):2247-76.
8. Ritchie ME, Phipson B, Wu D, Hu Y, Law CW, Shi W, et al. limma powers differential expression analyses for RNA-sequencing and microarray studies. *Nucleic Acids Res.* 2015;43(7):e47.
9. Hahne F, and Ivanek R. Visualizing Genomic Data Using Gviz and Bioconductor. *Methods Mol Biol.* 2016;1418:335-51.

10. Ranathunge C, Patel SS, Pinky L, Correll VL, Chen S, Semmes OJ, et al. promor: a comprehensive R package for label-free proteomics data analysis and predictive modeling. *Bioinform Adv.* 2023;3(1):vbad025.
