# Supplementary material for: Vascular smooth muscle cell–derived KIF13B inhibits proinflammatory responses to protect against atherosclerosis
Source: J Clin Invest. 2026 Jan 29;136(6):e194175. doi: 10.1172/JCI194175 (PMC12987658; doi:10.1172/JCI194175)

Full unedited gel for Figure 1B

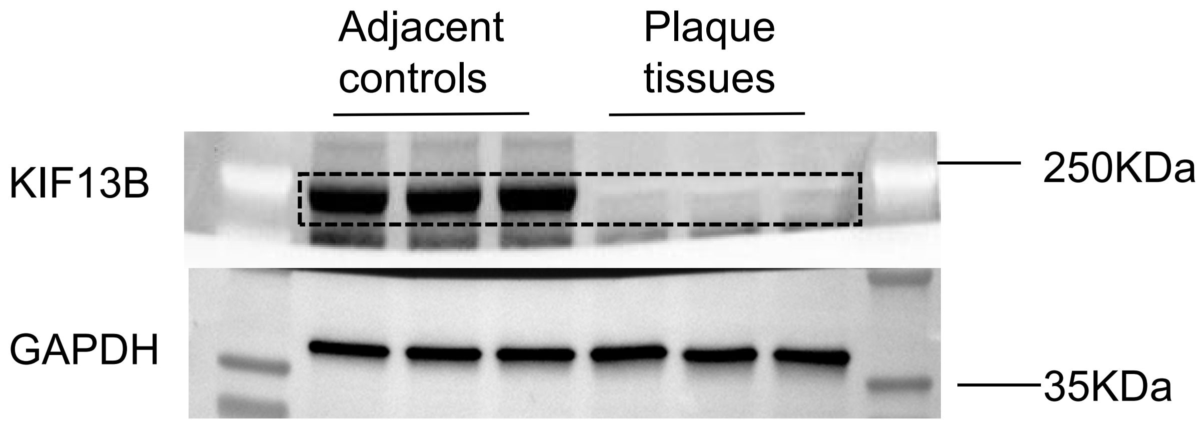

Full unedited gel for Figure 5L

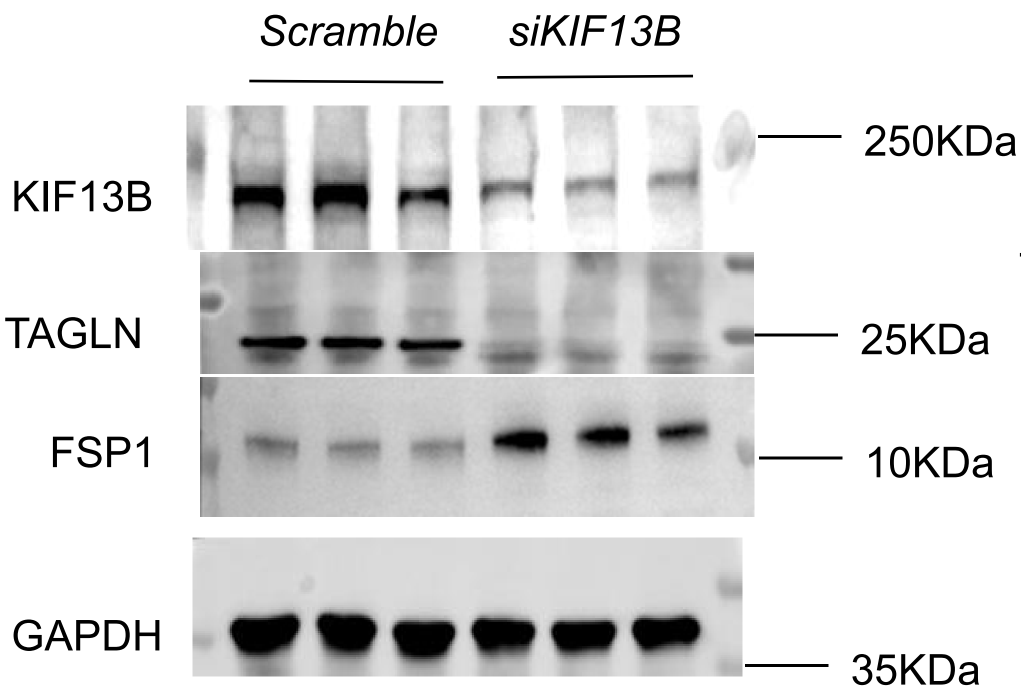

Full unedited gel for Figure 5O

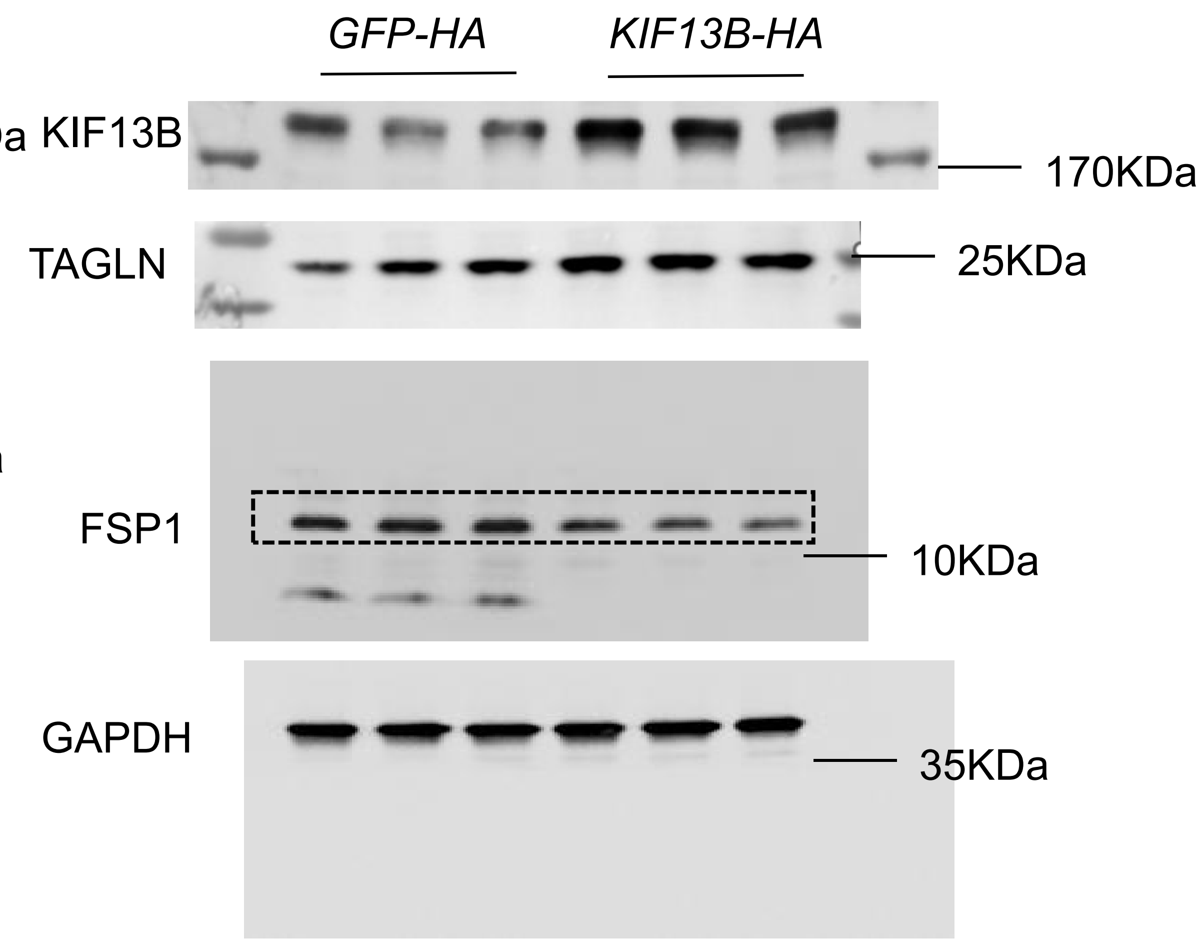

Full unedited gel for Figure 6C

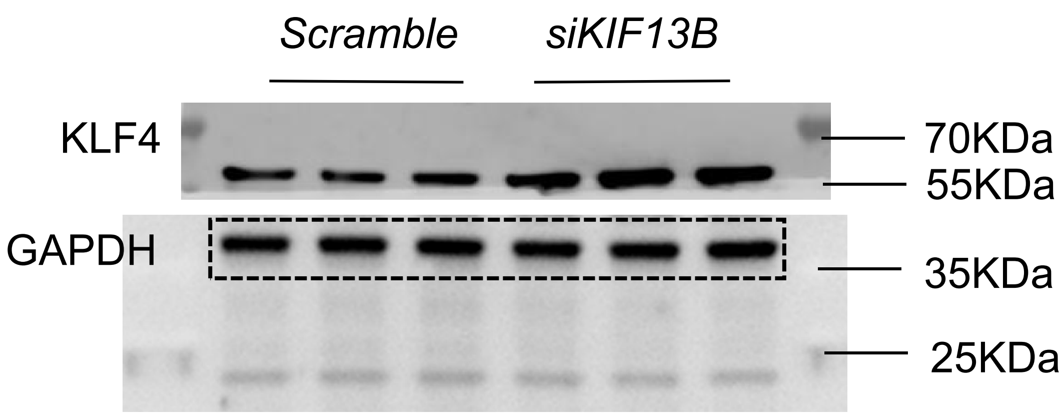

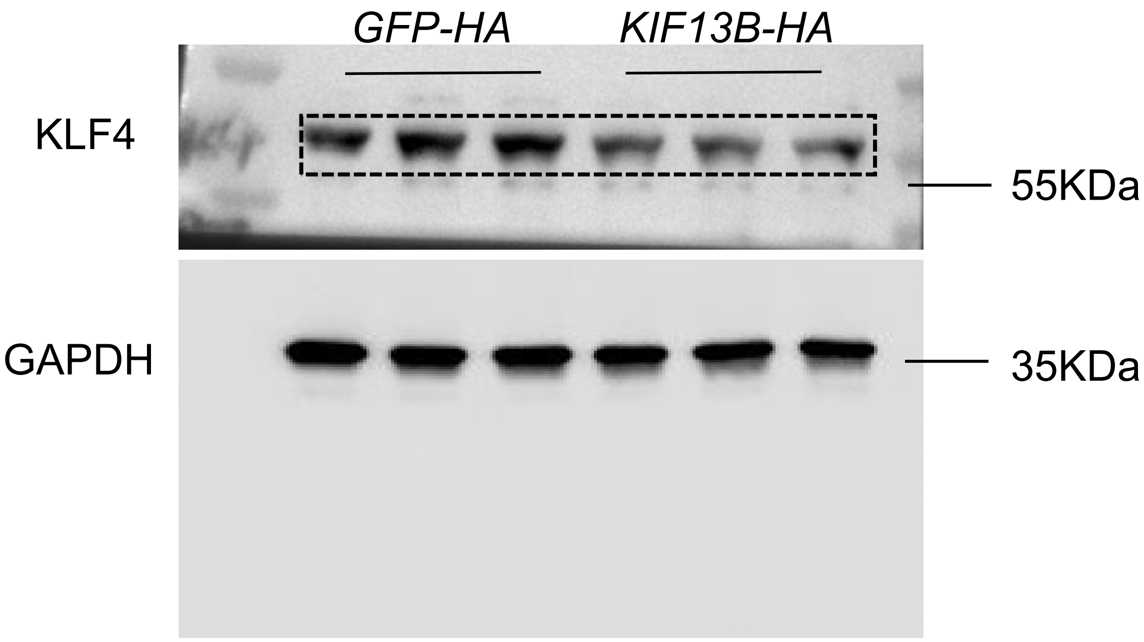

Full unedited gel for Figure 6F

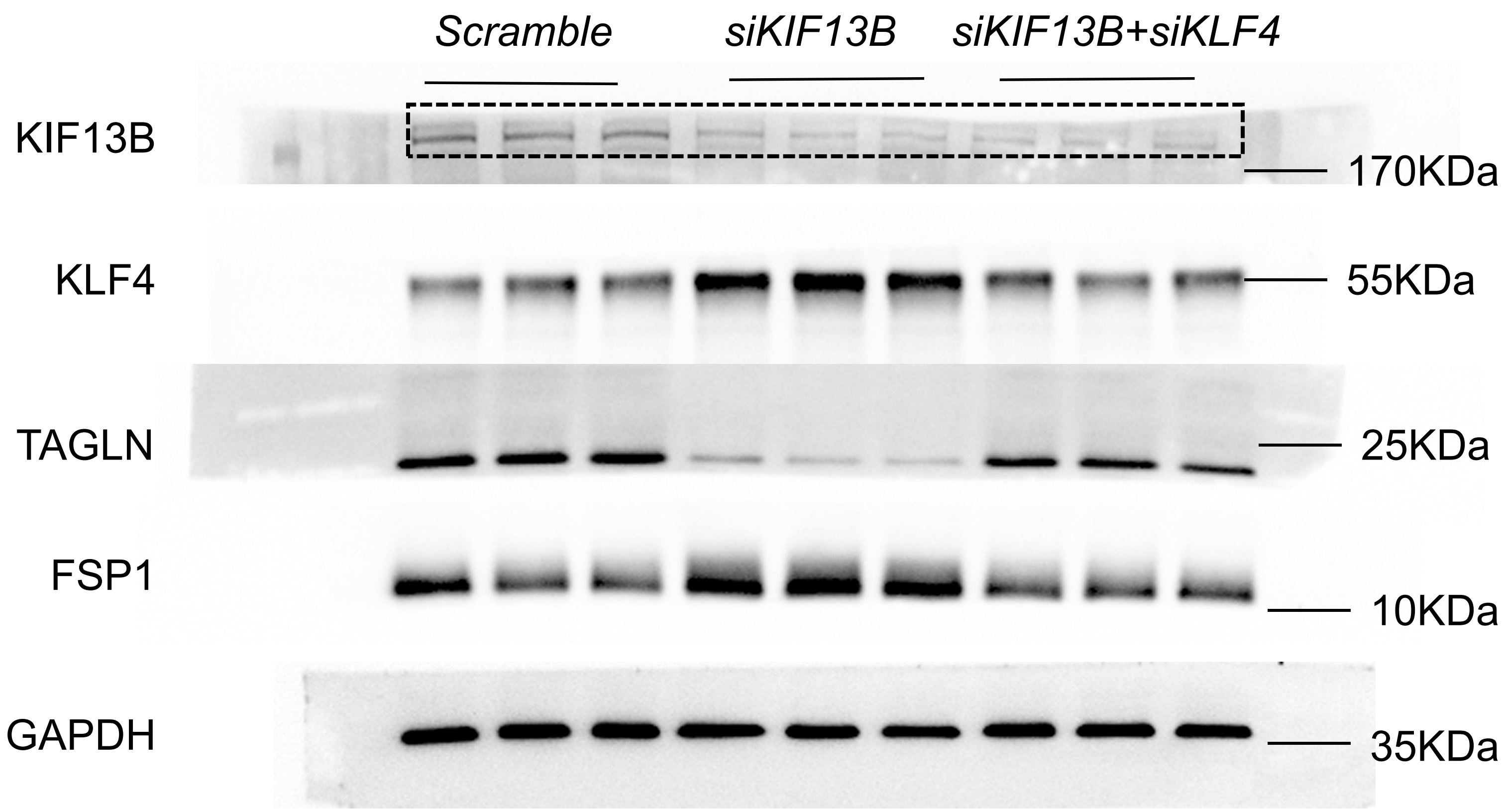

Full unedited gel for Figure 7A

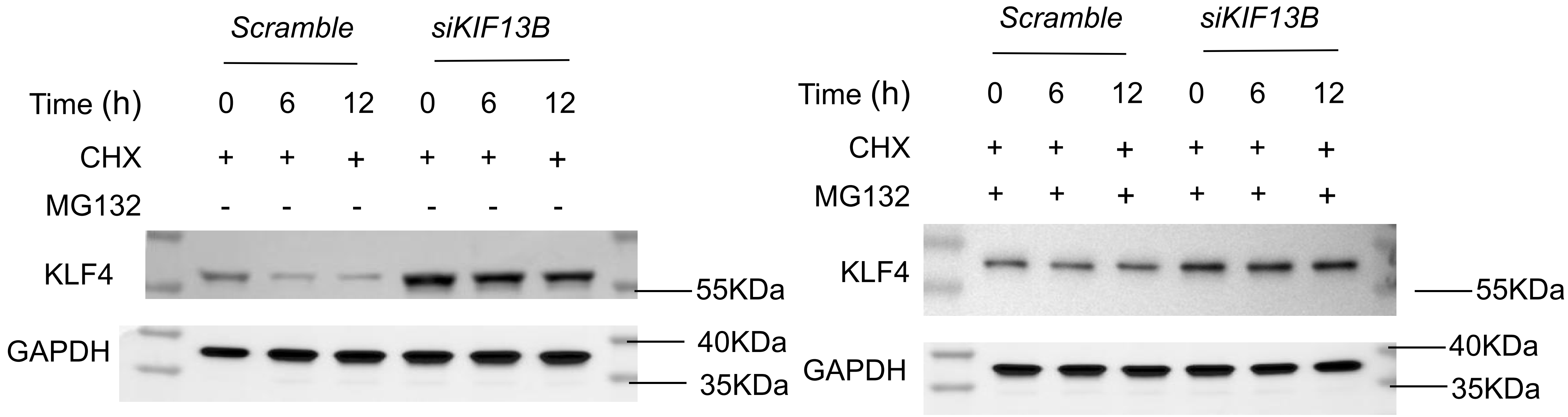

Full unedited gel for Figure 7B

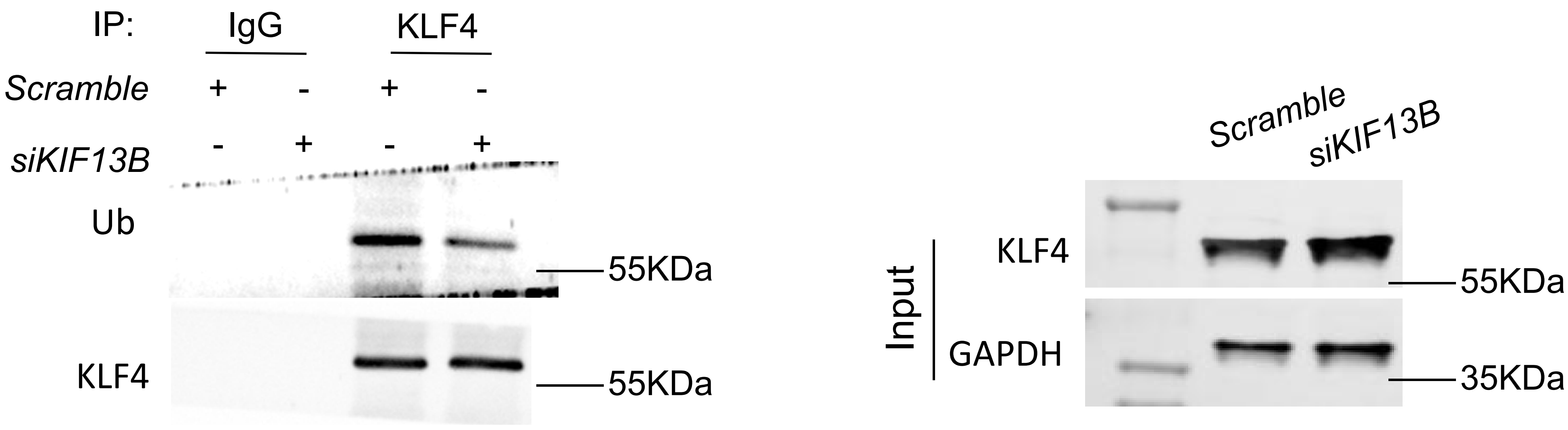

Full unedited gel for Figure 7D

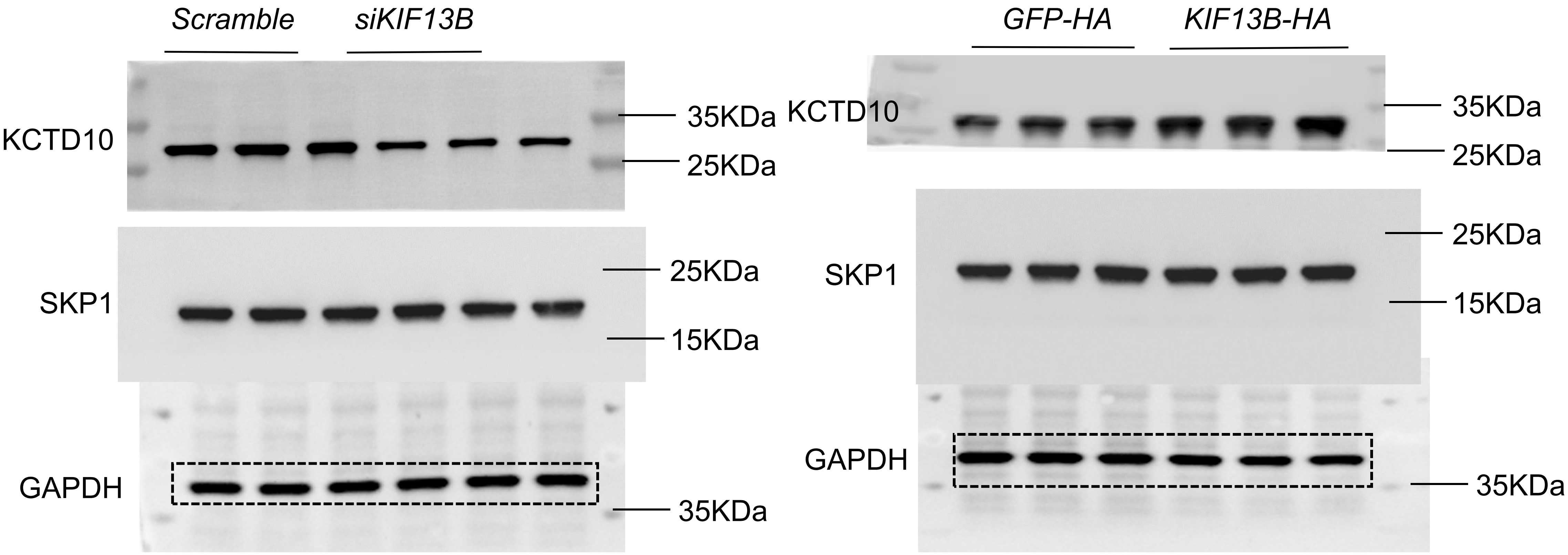

Full unedited gel for Figure 8B

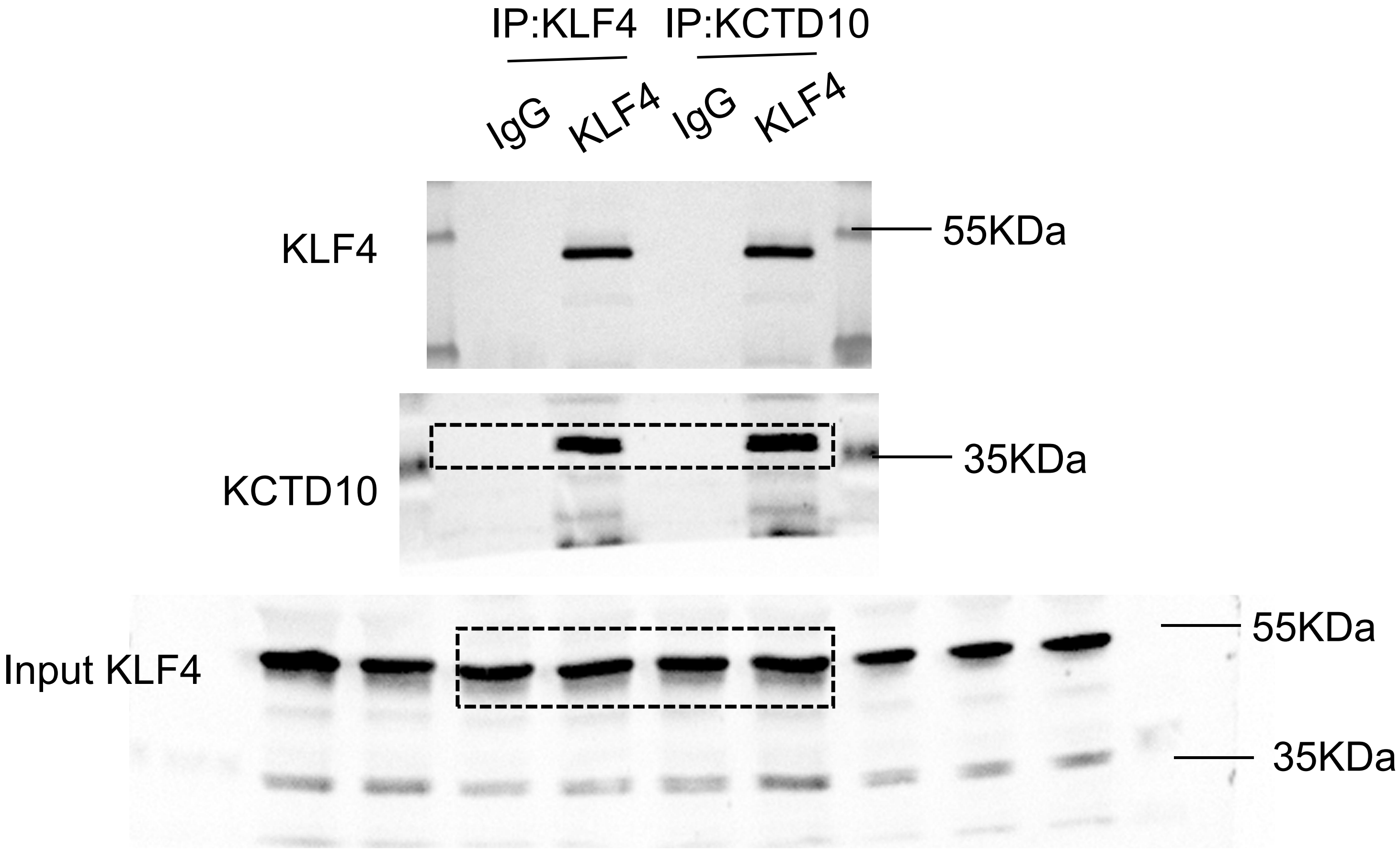

Full unedited gel for Figure 8C

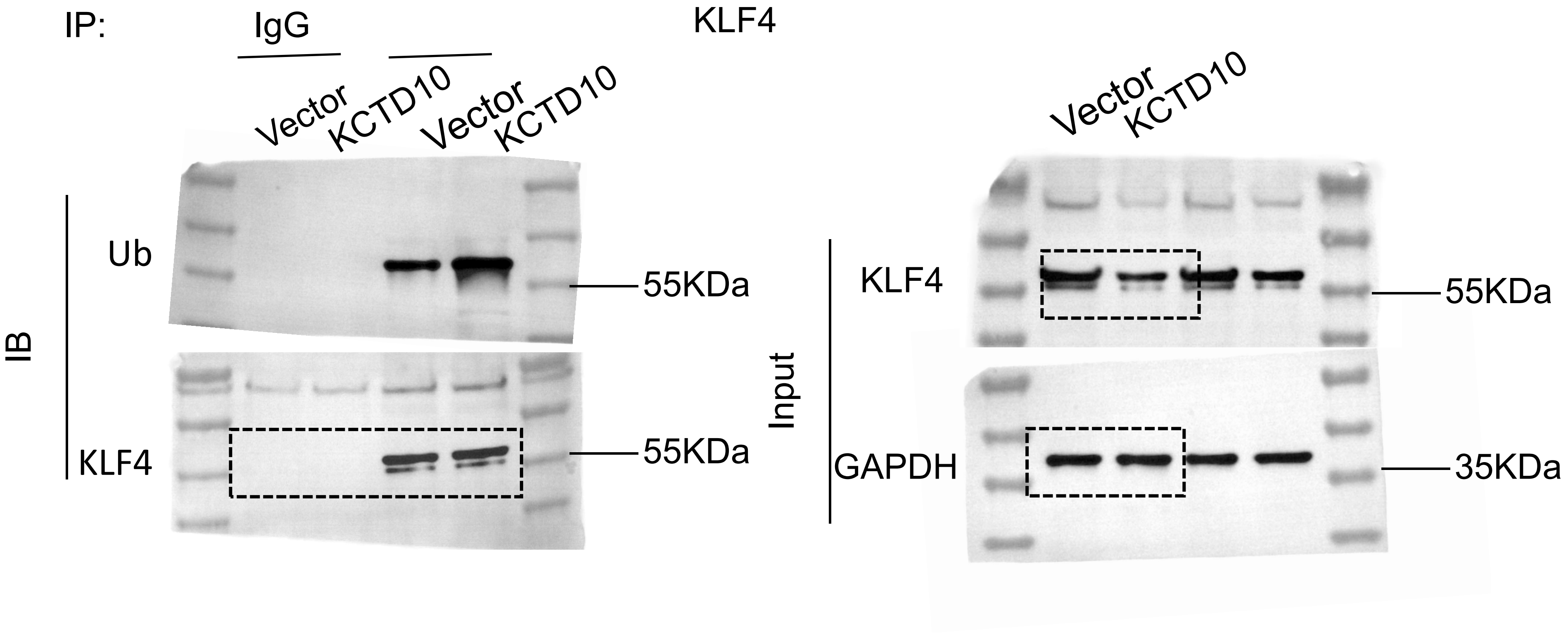

Full unedited gel for Figure 8D

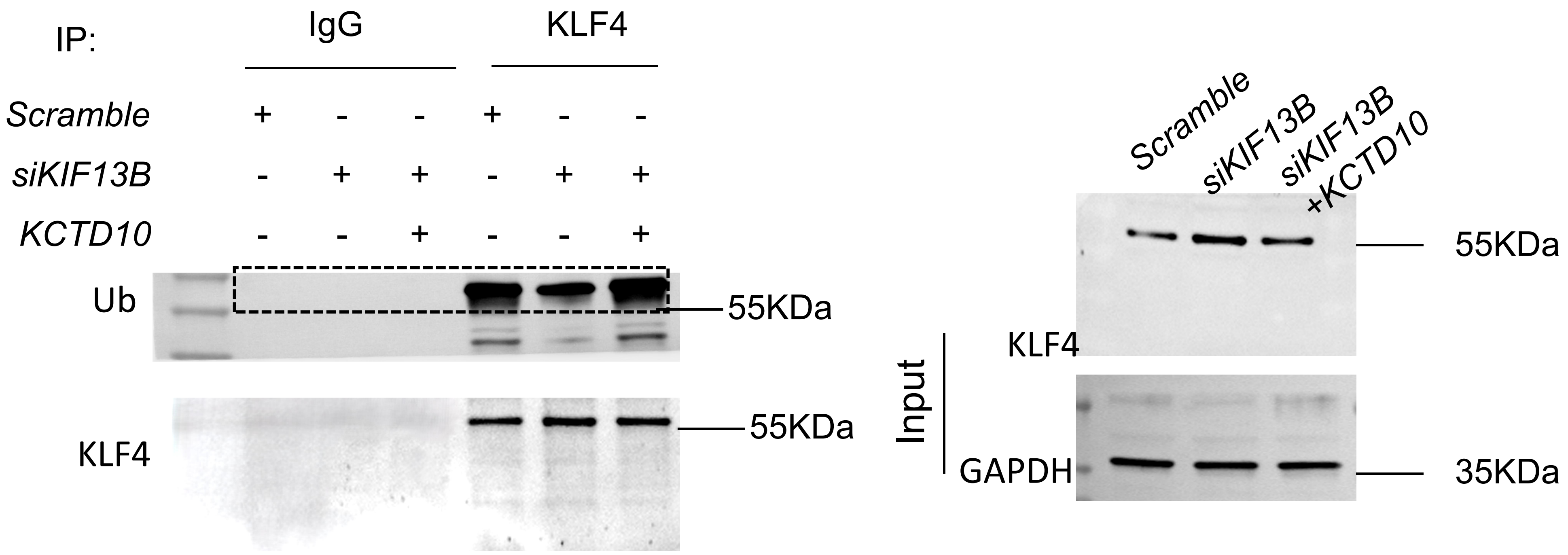

Full unedited gel for Figure 8E

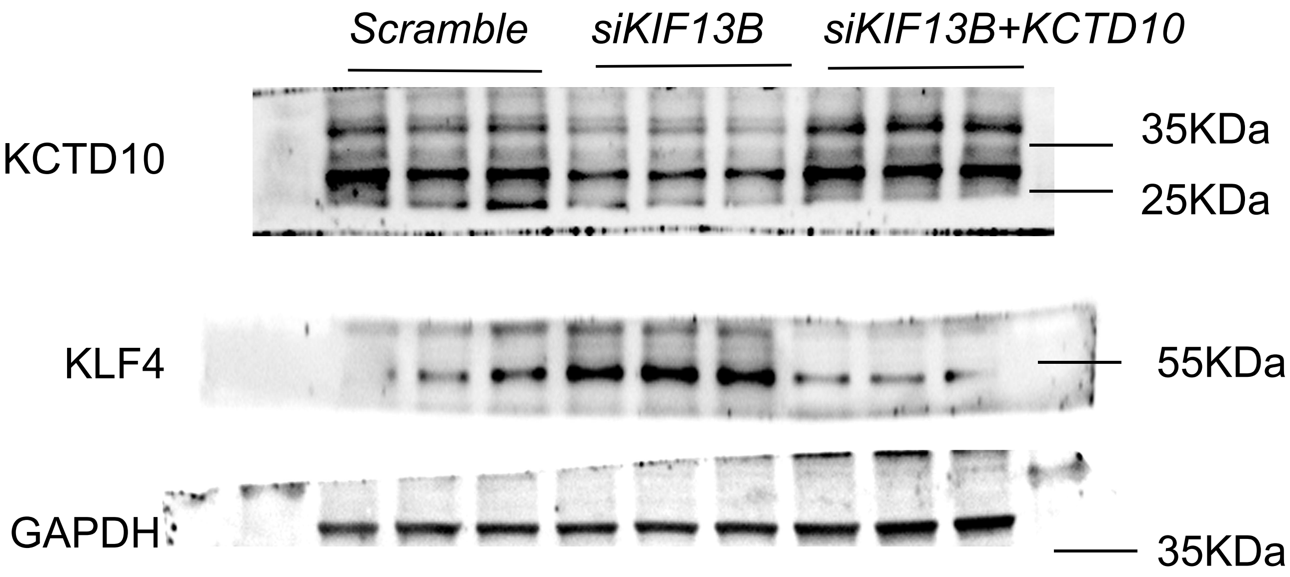

Full unedited gel for Figure 9B

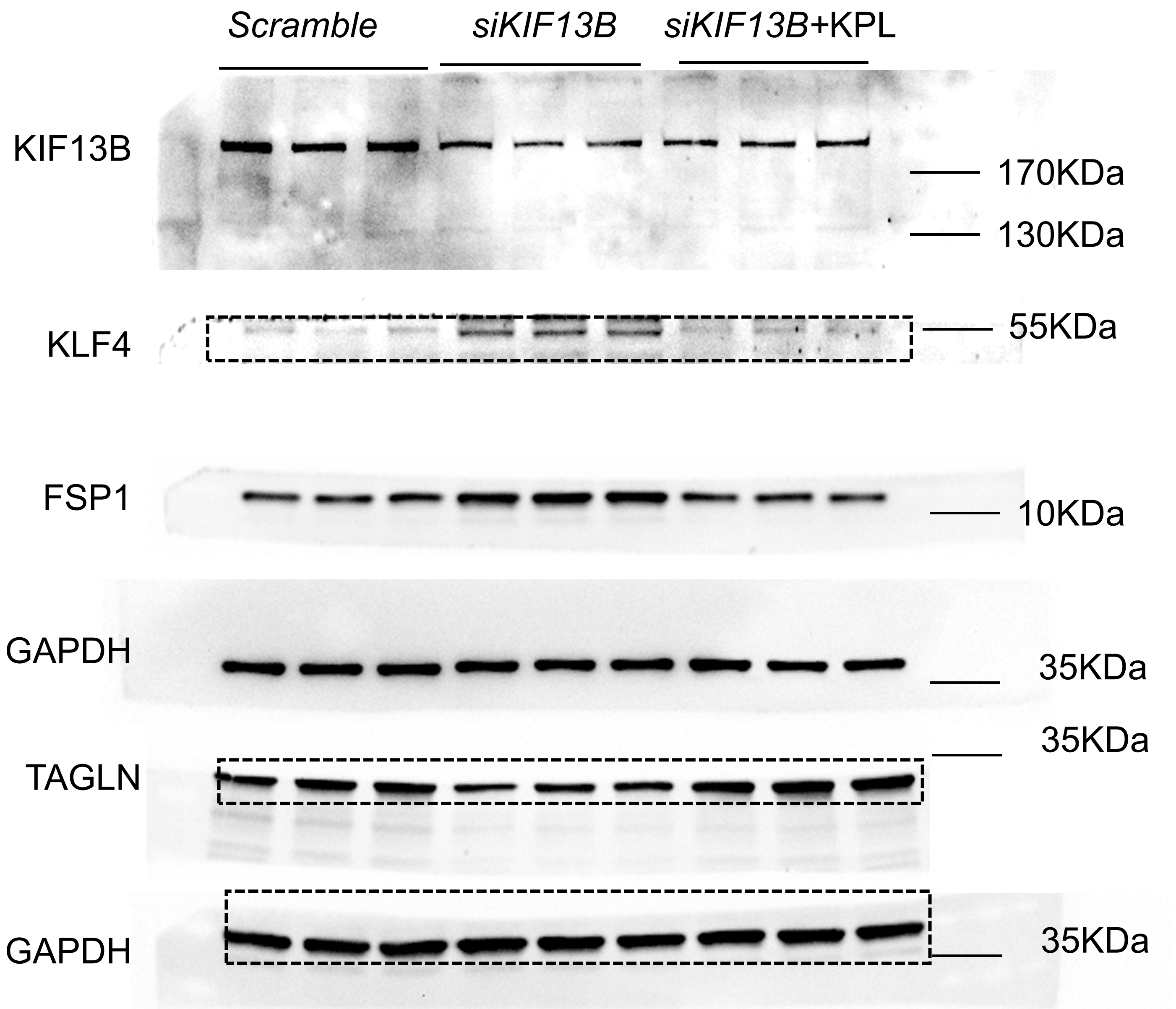

Supplement: Unedited blot and gel images [file jci-136-194175-s269.pdf]
